# Pck1 Deficiency Drives Mitochondrial Dysfunction and Cellular Senescence in Adipocytes

**DOI:** 10.1111/acel.70462

**Published:** 2026-03-30

**Authors:** Yiting Lei, Meng Yang, Xiaoyun Jiang, Yujie Zhang, Yuzhi Chen, Weiheng Xie, Qihui Dai, Weihong Qin, Xiuqin Deng, Xiaojun Zhang, Zhongjun Zhou, Gonghua Huang, Xinguang Liu

**Affiliations:** ^1^ Guangdong Provincial Key Laboratory of Medical Immunology and Molecular Diagnostics, Institute of Aging Research, School of Medical Technology Guangdong Medical University Dongguan China; ^2^ School of Biomedical Sciences, LKS Faculty of Medicine The University of Hong Kong Hong Kong China

**Keywords:** aging, cellular senescence, cGAS/STING signaling, Pck1, TCA cycle, white adipose tissue

## Abstract

Cellular senescence of white adipose tissues (WAT) represents an early hallmark of aging; however, the involved mechanisms remain incompletely understood. Here, we identified the cytosolic phosphoenolpyruvate carboxykinase (Pck1) as a key regulator of mitochondrial function and inflammaging in WAT. Pck1 expression was downregulated in both gonadal WAT and inguinal WAT during aging, and adipocyte‐specific Pck1 deficiency accelerated inflammaging and metabolic disorders. Untargeted metabolomic and isotope‐tracing analyses revealed that loss of Pck1 impaired cataplerosis, the export of tricarboxylic acid (TCA) cycle intermediates, resulting in accumulation of fumarate in adipocytes. Supplementation with exogenous fumarate disrupted mitochondrial homeostasis of adipocytes, promoted oxidative stress and triggered cytosolic release of mitochondrial DNA (mtDNA), leading to the activation of the cyclic GMP‐AMP synthase/stimulator of interferon genes (cGAS/STING) signaling pathway that may contribute to inflammaging and chronic obesity. These were phenocopied with Pck1‐deficient adipocytes. Conversely, overexpression of fumarate hydratase (Fh1) reduced fumarate level substantially and attenuated adipocyte inflammaging. Collectively, these findings identify Pck1 as a pivotal regulator of mitochondrial metabolic homeostasis and suggest that targeting Pck1 may represent a promising therapeutic strategy for age‐related diseases.

## Graphical abstract

Phosphoenolpyruvate carboxykinase 1 (Pck1) expression is downregulated in aging white adipose tissue. Adipocyte Pck1 deficiency impairs TCA cycle cataplerosis, causing fumarate accumulation. Excess fumarate disrupts mitochondrial homeostasis, triggers oxidative stress and mtDNA release, activating cGAS–STING signaling and driving inflammaging.

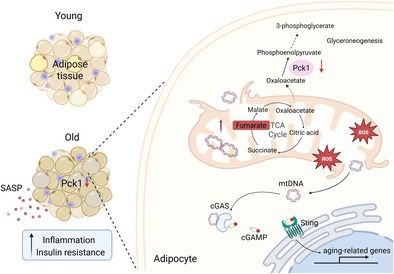

## 1 Introduction

Aging is a multifaceted and inevitable process characterized by a progressive decline in cellular functionality and deterioration of tissues and organs, making individuals more susceptible to multiple age‐related diseases (Nguyen and Corvera 2024; Kroemer et al. 2025). Substantial evidence demonstrates that aging and age‐related disorders are closely linked to adverse metabolic outcomes, and considerable progress has been achieved in extending the healthy lifespan by caloric restriction and exercise to ameliorate metabolic abnormalities (Mutlu et al. 2021; Johnson and Stolzing 2019; López‐Otín et al. 2016). However, the precise molecular mechanisms by which metabolic abnormalities affect the hallmarks of aging remain largely unresolved.

Adipose tissue is one of the most vulnerable tissues during aging, and its dysfunction plays a pivotal role in age‐associated physiological impairments (Ou et al. 2022). As the primary energy storage and endocrine organ, adipose tissue serves as a central hub for maintaining energy and metabolic homeostasis (Zwick et al. 2018). Age‐related accumulation of the dysfunctional adipose tissue, particularly visceral fat, induces chronic inflammation and insulin resistance, leading to metabolic disorders (Reyes‐Farias et al. 2021; Zhang, Jiang, et al. 2024). Clearance of senescent cells in white adipose tissue (WAT) has been shown to significantly mitigate aging‐related metabolic dysfunction in murine models, highlighting a pivotal role of WAT in organismal aging (Palmer et al. 2019; de Oliveira et al. 2025).

Senescent cells undergo metabolic reprogramming, resulting in abnormal accumulation of detrimental metabolites that further reinforce inflammatory and senescence pathways (Maqdasy et al. 2022; Dou et al. 2023; Zhang, Higgins, et al. 2024). These cells are identified by a combination of molecular features, including elevated senescence‐associated β‐galactosidase (SA‐β‐gal) activity, increased expression of cyclin‐dependent kinase inhibitors p21 and p16, persistent DNA damage response, and secretion of the senescence‐associated secretory phenotype (SASP) (Di Micco et al. 2021). Mitochondrial dysfunction is a critical driver of cellular senescence (Gonzalez‐Freire et al. 2015), marked by impaired mitochondrial quality control, heightened oxidative stress, accumulation of mitochondrial DNA damage, and disruption of cytosolic nicotinamide adenine dinucleotide (NAD^+^ and NADH) balance (de Mello et al. 2018; Das et al. 2021). Emerging studies indicate that impaired TCA cycle metabolism reduces mitochondrial membrane potential, exacerbates mitochondrial damage, and contributes to cellular senescence (Kurhaluk 2024; Cappel et al. 2019). However, the precise mechanisms linking mitochondrial dysfunction to the onset and progression of cellular senescence and age‐related inflammation remain incompletely understood.

Phosphoenolpyruvate carboxykinase 1 (Pck1) is well recognized as a rate‐limiting enzyme in hepatic and renal gluconeogenesis; however, in adipose tissue, it predominantly facilitates glyceroneogenesis, playing a pivotal role in modulating mitochondrial‐cytosolic carbon flux via TCA cycle cataplerosis. Our prior research demonstrates that Pck1 modulates replicative lifespan in yeast (Yuan et al. 2020), suggesting its potential role in aging processes. In this study, we showed that Pck1 was essential for mitochondrial function and adipose tissue aging. Pck1 deficiency induced premature adipocyte senescence, exacerbated age‐related metabolic abnormalities and inflammation in WAT. Mechanistically, Pck1 deficiency impaired TCA cycle catabolism, leading to fumarate accumulation, which further promoted cellular senescence. These findings identify Pck1 as a key metabolic effector that links mitochondrial metabolism in adipocytes to adipocyte aging and inflammation, with potential implications for targeting age‐related diseases.

## 2 Results

### 2.1 Pck1 Expression Is Decreased in WAT During Aging Process

**FIGURE 1:**
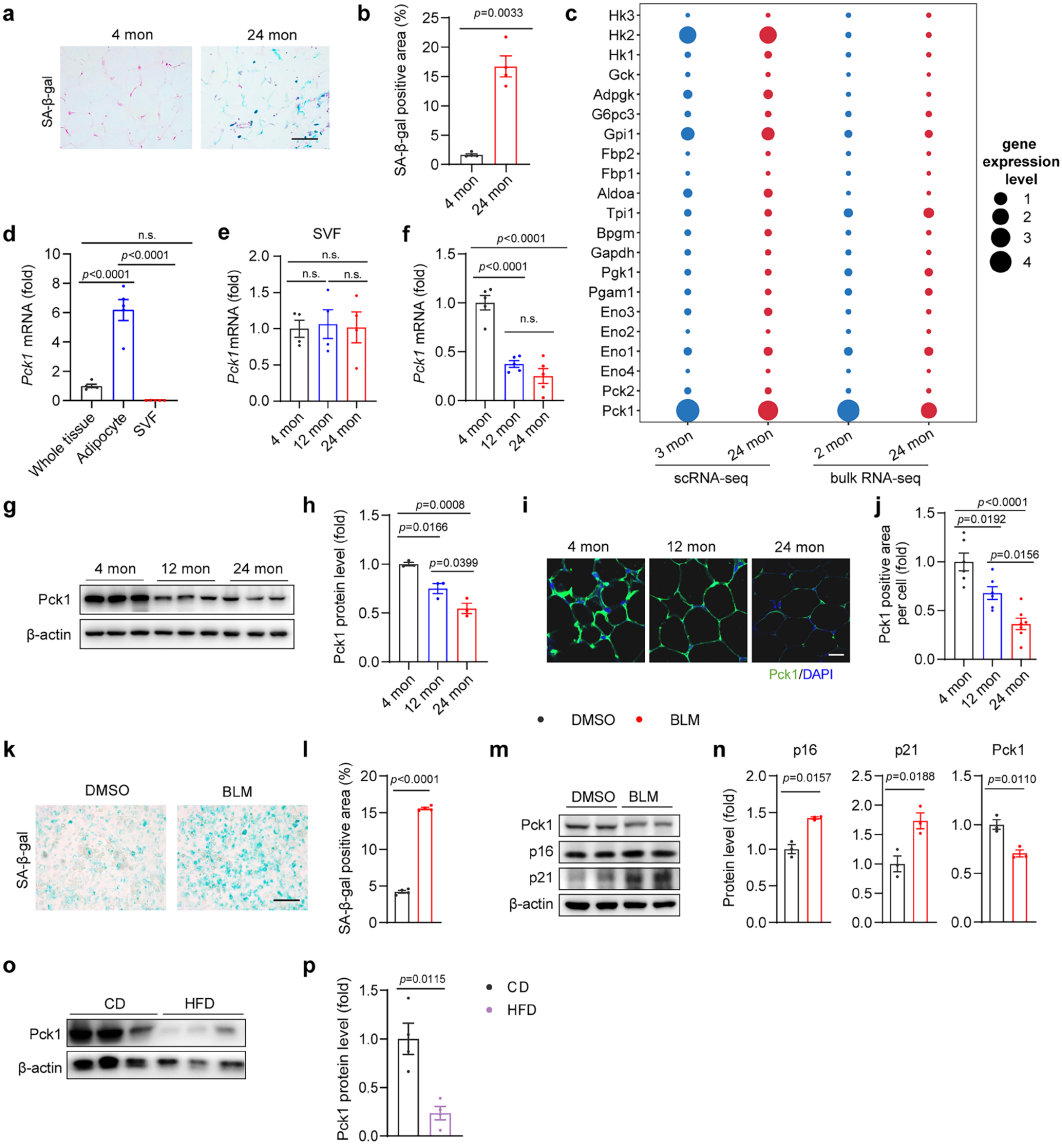
Pck1 expression in adipocytes is decreased during aging. (a) Representative images of SA‐β‐gal staining on gWAT of 4‐month‐old or 24‐month‐old C57BL/6 mice (scale bar, 50 μm). (b) Quantitative analysis of total SA‐β‐gal positive area (*n* = 4). (c) Expression of genes linked to the glyceroneogenesis pathway in white adipocytes of young mice (blue) and old mice (red). The size of bubbles indicates average gene expression within respective groups. Information was retrieved from the NCBI GEO database (GSE247719) or C57BL/6 mice RNA‐seq. (d) RT‐qPCR detected the relative transcript level of Pck1 in different organizational components with 4‐month‐old WT mice (*n* = 5). (e) RT‐qPCR detecting the Pck1 mRNA expression in SVFs of WT mice across three age groups. (f) RT‐qPCR analysis of Pck1 mRNA level during the process of aging in gWAT of C57BL/6 mice (*n* = 5). (g, h) Western blot analysis of Pck1 protein levels of gWATs during aging, β‐actin, loading control (*n* = 3). (i) Immunofluorescence images of Pck1 expression in different states of aging. (j) Quantitative analysis of total fluorescence intensity (*n* = 6) (Scale bar, 20 μm). (k, l) SA‐β‐gal staining and quantitative analysis of positive area in SVF‐derived adipocytes which were treated with BLM or DMSO (*n* = 4) (Scale bar, 200 μm). (m, n) Western blot analysis of p16, p21, and Pck1 in BLM‐induced senescent white adipocytes. (o, p) Western blot analysis of Pck1 in gWATs of CD or HFD WT mice (*n* = 4). β‐Actin, loading control. Data are shown as the mean ± SEM for all panels. Data were analyzed by two‐tailed unpaired *t* test (b, l, n, p) or one‐way ANOVA, followed by Tukey's post hoc tests (d‐f, h, j).

To examine the impact of aging on glucometabolic gene expression in adipose tissue, we collected gonadal WAT (gWAT) and inguinal WAT (iWAT) from three age groups: young (2 or 4 months), middle‐aged (12 months), and old (24 months). SA‐β‐gal staining of young and aged gWAT samples confirmed that advanced age promoted adipose tissue senescence (Figure 1a,b). We examined 21 genes involved in gluconeogenesis/glyceroneogenesis or glycolysis and integrated their expression with data from the adipocyte subset of gWAT scRNA‐seq (GEO: GSE247716) and bulk RNA‐seq data from gWAT samples of young and aged mice. Unexpectedly, this cross‐referencing showed that Pck1 was highly expressed in adipocytes and whole gWAT with significant downregulation in aged mice (Figure 1c). RT‐qPCR analysis showed that Pck1 expression was predominantly restricted to mature adipocytes, with minimal expression in the stromal vascular fraction (SVF) components in 4‐month‐old wild‐type (WT) mice (Figure 1d). Furthermore, we found that both gWAT and iWAT exhibited a significantly age‐dependent reduction in Pck1 expression at both the mRNA and protein levels, but this was not observed in SVF components (Figure 1e–j; Figure S1a,b). To further validate these findings in vitro, we induced DNA damage response‐mediated senescence using bleomycin (BLM) in adipocytes derived from SVFs and found that Pck1 expression was also diminished in senescent adipocytes (Figure 1k–n). Additionally, we assessed Pck1 expression in a model of obesity‐driven accelerated aging. Our results showed that high‐fat diet (HFD) feeding significantly decreased Pck1 expression compared to chow diet (CD) (Figure 1o,p). Together, these results demonstrate that aging leads to a consistent decline in Pck1 expression in white adipocytes.

### 2.2 Adipocyte‐Specific Ablation of Pck1 Promotes Premature Pathological Senescence and Age‐Related Metabolic Disorders

**FIGURE 2:**
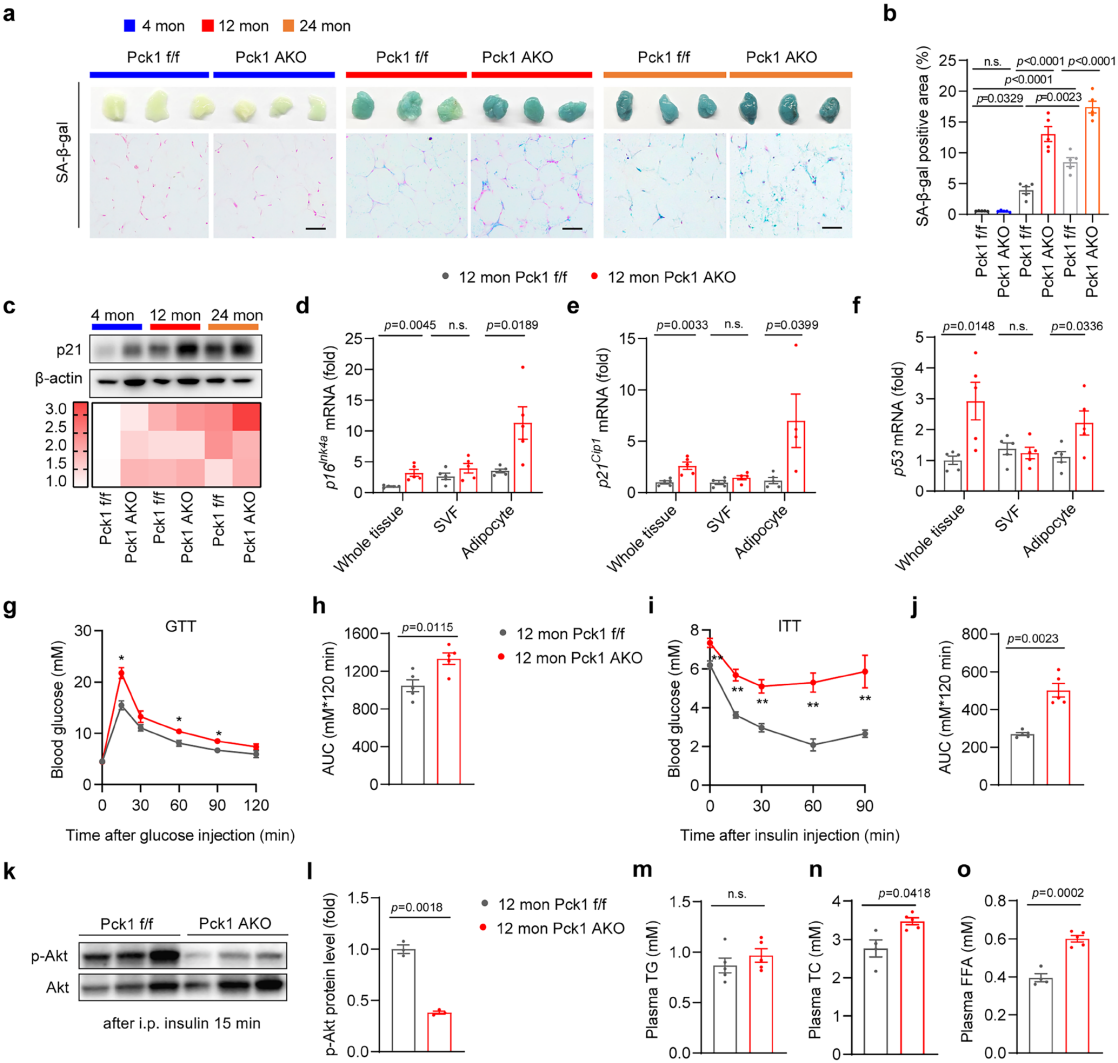
Adipocyte‐specific Pck1 ablation aggravates gWAT cellular senescence and metabolic disorder. (a) Representative images of SA‐β‐gal staining of gWAT in 4‐, 12‐, and 24‐month‐old Pck1 AKO and control mice (scale bar, 50 μm). (b) Quantification of SA‐β‐gal‐positive area from individual images (*n* = 5). (c) Western blot analysis of senescence‐related protein in adipose tissues (*n* = 3). Protein levels are displayed as a heatmap at the bottom. (d–f) Senescence‐related mRNA expression in sub‐organizational components from gWAT (*n* = 4). (g, h) GTT and ITT were performed on 12‐month‐old Pck1 AKO and Pck1 f/f (control) mice (*n* = 5). (i, j) The AUC data for GTT and ITT were calculated. (k) Western blot images of Akt phosphorylation after i.p. inject 1 U/kg insulin for 15 min. (l) Quantification of Akt phosphorylation relative grayscale values normalize to total Akt (*n* = 3). (m–o) Plasma TG, TC, and FFA levels of Pck1 f/f and AKO mice under 16 h fasting condition (*n* = 5). Data are presented as the mean ± SEM and analyzed by two‐way ANOVA with Tukey's post hoc analysis (b), and two‐tailed unpaired Student's *t*‐tests (d–o). * FDR < 0.05 and ** FDR < 0.01 by *t*‐test with Benjamini‐Hochberg FDR correction for multiple comparisons.

To investigate the potential role of Pck1 in WAT aging, we generated adipocyte‐specific Pck1 knockout (Pck1 AKO) mice by crossing Adiponectin‐cre (Adipoq‐cre) with Pck1 f/f mice. At 6 weeks of age, histological analysis revealed no significant differences in the kidneys, liver, spleen, or other major organs between Pck1 f/f and Adipoq‐cre mice (Figure S4a). Sex‐matched littermate Pck1 f/f mice served as controls for Pck1 AKO animals. Compared to Pck1 f/f littermates, Pck1 AKO mice exhibited almost complete absence of Pck1 protein in both WAT and BAT (Figure S2a). Adipose tissues in middle‐aged mice have been shown to display transcriptomic features of aging (Schaum et al. 2020). We consistently observed an increased SA‐β‐gal staining positive area in gWAT and iWAT of middle‐aged and aged mice (Figure 2a,b; Figure S1c,d). Furthermore, Pck1 deficiency led to a significantly upregulated expression of certain senescence markers in WAT of Pck1 AKO mice compared to Pck1 f/f mice (Figure 2c; Figure S1g). To further examine the primary cell types in adipose tissue most susceptible to Pck1 deficiency–induced senescence, we conducted RT‐qPCR analysis on mature adipocytes and SVFs isolated from gWATs of 12‐month‐old Pck1 AKO and control mice. Our results revealed a selective upregulation of senescence‐associated genes, including *p16* ^*Ink4a*^, *p21* ^*Cip1*^, and *p53*, in mature adipocytes from Pck1 AKO mice, whereas expression of these transcripts in the SVF remained comparable to controls with no significant differences (Figure 2d–f), and the expression of the above genes was gradually increased during aging (Figure S2b). These results indicate that Pck1 deficiency promotes adipocyte senescence.

Dysfunction of aged adipose tissue contributes to the disruption of lipid and glucose homeostasis (Morigny et al. 2021; Von Bank et al. 2021). To elucidate the role of *Pck1* deletion in adipocytes in age‐related metabolic disorders, we performed glucose tolerance tests (GTT) and insulin tolerance tests (ITT) across various age groups. We first observed that normal aged mice exhibited impaired glucose tolerance and elevated plasma levels of total cholesterol (TC) and free fatty acids (FFA) (Figure S2c–e). Compared to Pck1 f/f mice, Pck1 AKO mice displayed a slightly increased blood glucose at 4 months old (Figure S3a,b). However, 12‐month‐old and 24‐month‐old Pck1 AKO mice displayed significantly impaired glucose tolerance and increased insulin resistance (Figure 2g–j; Figure S3c,d). Moreover, Pck1 deficiency impaired adipose tissue insulin signaling in vivo, as evidenced by reduced phosphorylation of AKT following intraperitoneal insulin administration (Figure 2k,l). Furthermore, 12‐month‐old Pck1 AKO mice had significantly elevated plasma levels of TC and FFA compare to the WT controls (Figure 2m–o). Similarly, 24‐month‐old mice had significantly increased plasma FFA and triglycerides (TG), although TC levels were not statistically altered (Figure S3e). Notably, blood lipid profiles of young Pck1 AKO mice (4‐month‐old) did not show significant changes compared with controls (Figure S3f). Collectively, these findings underscore the critical role of Pck1 in adipocytes in maintaining adipose tissue and systemic metabolic homeostasis.

### 2.3 Deficiency of Pck1 in Adipocytes Promotes WAT Inflammaging

**FIGURE 3:**
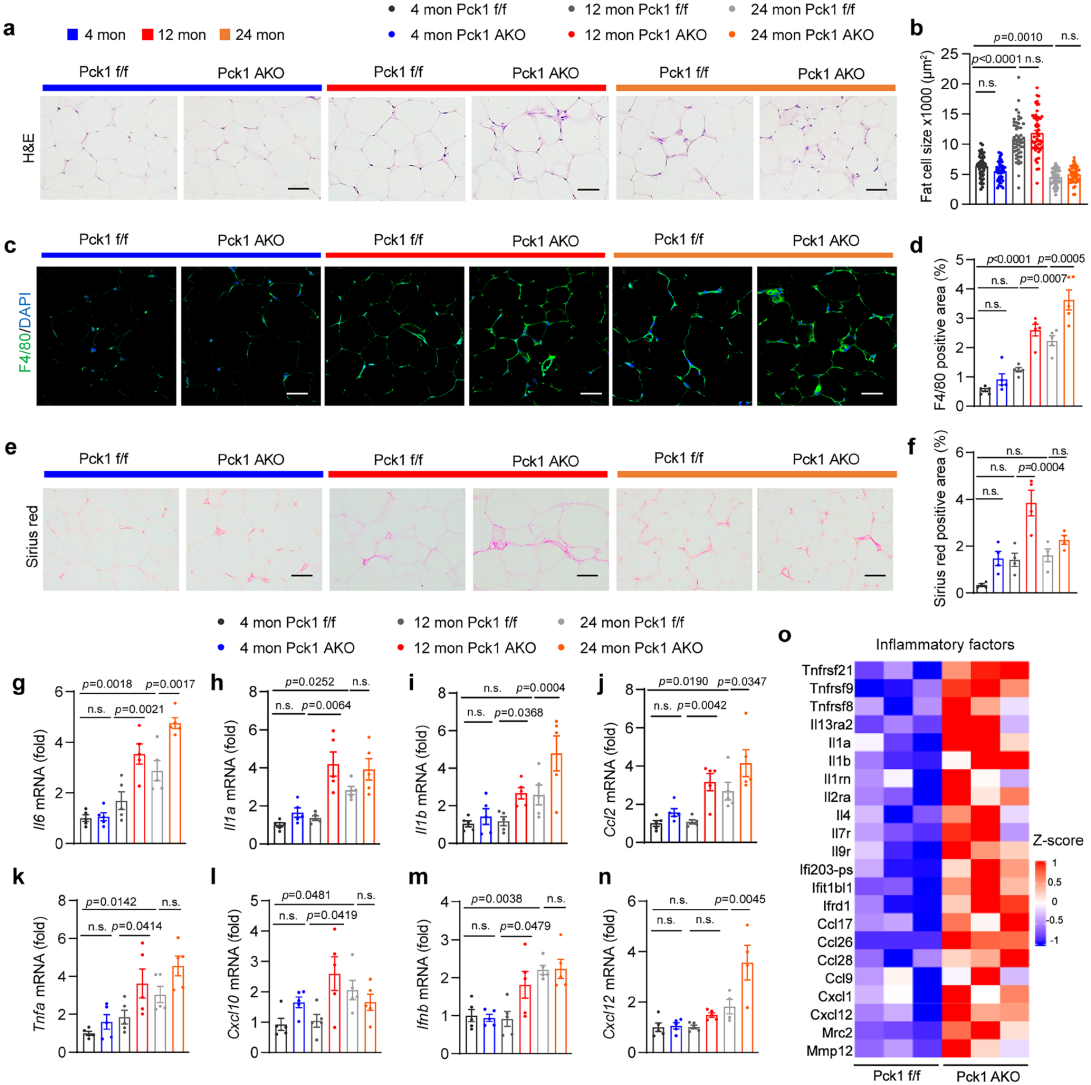
Deficiency of Pck1 in adipocytes promotes macrophage infiltration and SASP expression in gWAT. (a) Representative images of H&E staining of gWAT from the young, middle‐age, old groups (scale bars, 50 μm). (b) Quantitative analysis of the size of fat cells. Counted 10 cells per mouse (*n* = 5). (c) Representative immunofluorescence images of F4/80‐labeled (green) macrophages in gWAT with different groups (scale bars, 50 μm). (d) Quantification of F4/80 total fluorescence intensity (*n* = 5). (e) Representative images of adipose tissues with collagen staining (Sirius Red) (scale bars, 50 μm). (f) Quantification of Sirius red positive area percentage (*n* = 5). (g–n) RT‐qPCR analysis of SASP mRNA expression in gWAT from aged Pck1 f/f or Pck1 AKO mice. Relative to the young group (*n* = 5). (o) Heatmap to display upregulated SASPs in gWAT of 6‐month‐old Pck1 AKO mice. Data are shown as the mean ± SEM for all panels. Data were analyzed statistically via two‐way ANOVA with Tukey's HSD post hoc test for multiple comparisons (b–n). Differentially expressed genes were identified based on |fold change| > 1.5 and false discovery rate (FDR) < 0.05 (o).

Given that adipose tissue has been shown to be central for metaflammation (Franceschi et al. 2018), we want to investigate whether Pck1 deficiency influences SASP production and age‐related immune cell infiltration in adipose tissue. H&E staining of gWAT did not show statistical significance in the size of adipocytes; however, there was more cell infiltration in Pck1 AKO mice than in WT mice (Figure 3a,b). We further employed F4/80 immunohistochemical analysis to identify a notable, age‐dependent increase of inflammatory macrophages in gWAT of Pck1 AKO mice compared to the age‐matched controls (Figure 3c,d). Interestingly, adipose tissue fibrosis, a hallmark of inflammation and aging, was predominantly exacerbated in iWAT of Pck1 AKO mice compared to the age‐matched controls (Figure 3e,f; Figure S1e,f). The chronic release of SASP factors from senescent cells is known to affect neighboring cells within the host microenvironment (Nakao et al. 2020; Wang et al. 2024). Analyses of SASP mRNA levels in WAT across different aging stages revealed a significant upregulation of pro‐inflammatory factors, including interleukin‐6 (IL‐6), interleukin‐1 alpha (IL‐1α), interleukin‐1 beta (IL‐1β), chemokine ligand 2 (Ccl2, also known as monocyte chemoattractant protein‐1), and tumor necrosis factor‐alpha (TNFα), in both middle and older age mice, with Pck1 AKO mice even higher (Figure 3g–k; Figure S1h–k). Furthermore, interferon‐gamma‐inducible protein 10 (Cxcl10) and interferon β were considerably upregulated during the middle‐aged phase, while C‐X‐C motif ligand 12 (Cxcl12) showed upregulation exclusively in elderly Pck1 AKO mice (Figure 3l–n). Transcriptome analysis of gWAT further revealed a broader increase in inflammatory factors (Figure 3o). These findings demonstrate that Pck1 deficiency in adipocytes upregulates pro‐inflammatory and interferon‐stimulated genes, consequently establishing a pro‐inflammatory immune microenvironment through paracrine and endocrine signaling that exacerbates inflammaging.

### 2.4 Pck1 Deficiency Exacerbates Chronic Obesity‐Induced Adipose Tissue Inflammaging

**FIGURE 4:**
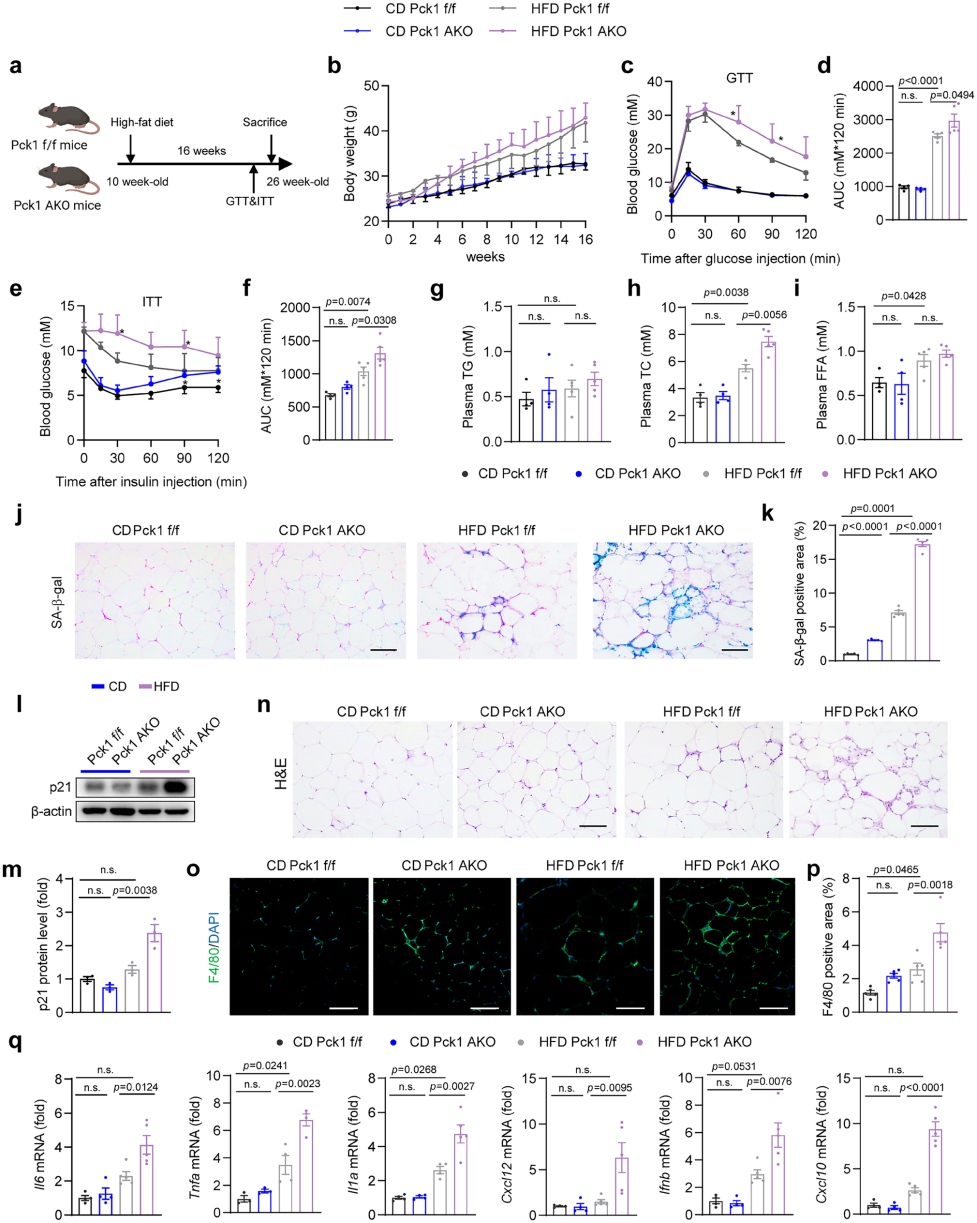
Deletion of Pck1 enhances adipocyte senescence, insulin resistance, and inflammation in a model of chronic obesity. (a) Schematic diagram depicting the experimental procedure for a long‐term HFD model. 10‐week‐old male Pck1 f/f and Pck1 AKO mice were randomized into two groups and fed either a CD or a HFD for 16 weeks. (Schematic created with BioRender.com. Agreement number: GA28ATR9M1). (b) Body weight curves of CD‐ and HFD‐fed mice. CD *n* = 4, HFD *n* = 5 biologically independent experiments. (c–f) GTT and ITT experiments were conducted within the last week of HFD, 6 days in between. (g‐i) Plasma TG, TC, and FFA levels of CD and HFD mice. (j, k) Representative images and quantitative analysis of SA‐β‐staining of gWAT (scale bars, 100 μm, *n* = 5). (l, m) Protein levels of p21 of gWAT from Pck1 f/f and Pck1 AKO mice fed with CD and HFD. (n) Representative images of H&E staining of CD and HFD mice (scale bars, 100 μm). (o) F4/80 immunofluorescence staining and quantitative analysis of gWAT (scale bars, 100 μm). (p) Quantitative analysis of F4/80 positive area (*n* = 5). (q) RT‐qPCR analysis of *Il6*, *Tnfa*, *Il1a*, *Cxcl12*, *Ifnb*, *Cxcl10* mRNA expression of gWAT from Pck1 f/f and Pck1 AKO mice at 16 weeks of CD or HFD (*n* = 4). Data are shown as the mean ± SEM for all panels. Statistical analysis is performed using two‐way ANOVA with Tukey's HSD post hoc analysis.

Obesity and aging shared common features such as chronic low‐grade inflammation in visceral adipose tissue (VAT), the development of metabolic diseases, and a shortened lifespan (Piché et al. 2020). To elucidate the precise role of Pck1 in an obesity‐induced aging model, we subjected 10‐week‐old Pck1 AKO mice and Pck1 f/f control mice to a 16‐week HFD feeding (Figure 4a). We observed that adipocyte‐specific *Pck1* deletion did not alter the body weight or in vivo metabolic phenotypes of CD‐fed mice (Figure 4b–i). However, despite no significant change in body weight (Figure 4b), HFD‐fed Pck1 AKO mice exhibited significantly impaired glucose tolerance and elevated insulin resistance, mirroring observations in aged mice (Figure 4c–f). Interestingly, the effect of Pck1 ablation on plasma lipids in obese mice was less pronounced compared to aged mice (Figure 4g–i).

We further assessed senescence markers in HFD‐fed Pck1 AKO mice and found that Pck1 deficiency led to senescent cell accumulation (Figure 4j,k) and increased p21 protein expression in gWAT (Figure 4l,m). As expected, increased immune cell infiltration was observed in the adipose tissue of HFD‐fed Pck1 AKO mice (Figure 4n). Moreover, macrophage infiltration (Figure 4n–p) and the expression of SASP factors, including *Il6*, *Tnfɑ*, *Il1a*, *Cxcl12*, *Ifnb*, and *Cxcl10*, were also increased in the gWAT of Pck1 AKO mice (Figure 4o–q). These findings reveal that Pck1 deficiency promotes gWAT senescence and inflammaging in the context of obesity and aging.

### 2.5 Pck1 Deficiency Leads to TCA Cycle Cataplerosis and Accumulation of Fumarate in Adipocytes

**FIGURE 5:**
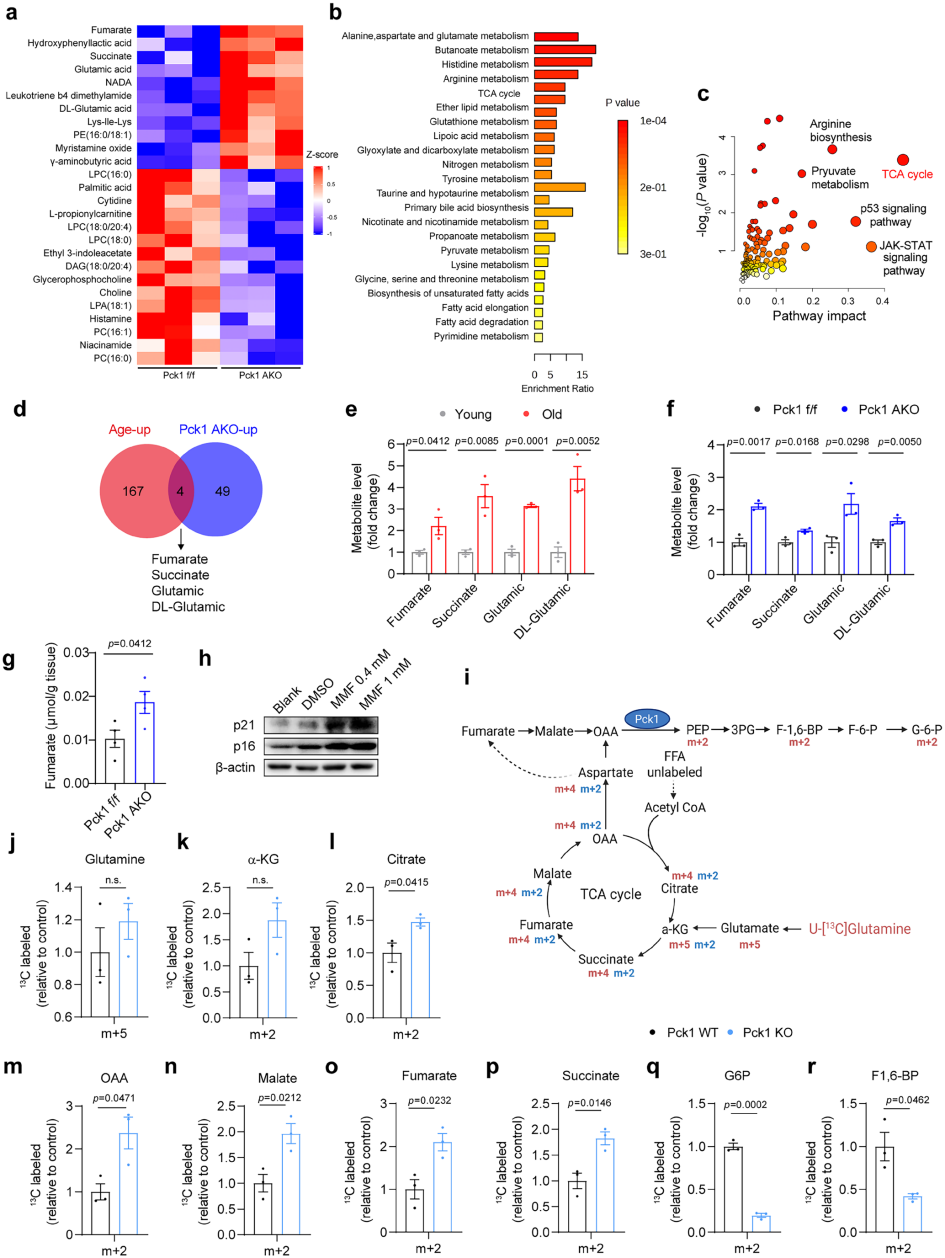
Pck1 deletion leads to fumarate accumulation to further exacerbate cellular senescence. (a, b) Global metabolic profiling of freshly obtained from 6‐month‐old mice Pck1 f/f and Pck1 AKO gWAT, heatmap with adjust *p* value < 0.05 and VIP > 1, followed by the Metabolite Set Enrichment Analysis (MESA) on https://www.metaboanalyst.ca. (c) Joint metabolomic and transcriptomic analyses were performed via MetaboAnalyst. Labels show the top 5 pathways with greatest significance and impact. (d) Venn diagram of dysregulated factors in the gWAT of mice (young versus aged or Pck1 f/f versus Pck1 AKO). (e, f) The fold changes of common metabolic intermediates in the metabolome. (g) Fumarate level analysis by LC–MS/MS in gWAT of 12‐month‐old Pck1 AKO and control mice (*n* = 5). (h) Protein levels of p16 and p21 in 3 T3‐L1 adipocytes treated with MMF or Vehicle. (i) Schematic diagram of Pck1 mediated metabolic reprogramming (Schematic created with BioRender.com. Agreement number: BJ28DKS2PJ). (j–r) LC–MS profiles of metabolites in Pck1 KO cells after incubation with U‐[^13^C]‐glutamine for 24 h (*n* = 3). Data are showed as mean ± SEM. Statistical analyses through the unpaired, two‐tailed Student's *t*‐test.

To further elucidate the regulatory mechanism of Pck1 in adipose tissue senescence, we performed untargeted metabolomics profiling of gWAT from Pck1 AKO and control mice. A total of 54 metabolites exhibited significant alterations between Pck1 AKO and control group, with upregulated metabolites predominantly consisting of organic acids and downregulated metabolites primarily comprising lipid metabolism intermediates (Figure 5a). Metabolome set enrichment analysis (MSEA) showed that Pck1 deficiency significantly upregulated the pathways related to amino acid, carbohydrate, and lipid metabolism (Figure 5b). Integrated transcriptomic and metabolomics analyses further identified the TCA cycle as the most significantly altered metabolic process in Pck1‐deficient adipose tissue (Figure 5c). Further analysis of all age‐associated metabolites in Pck1‐deficient adipose tissue entailed a comparative analysis of increased metabolites in metabolite profiles from aged (24‐month‐old) versus young (2‐month‐old) WT mice, alongside Pck1 AKO versus Pck1 f/f mice. This analysis identified four metabolites, including fumarate, succinate, glutamate, and DL‐glutamate, that were significantly upregulated in Pck1 AKO mice (Figure 5d–f). Strikingly, all these metabolites are intermediates of the TCA cycle—a finding that aligns with observations in senescent MEF cells, aged mouse brains and aged *C. elegans* (Dou et al. 2023; Ding et al. 2021; Wan et al. 2017). LC–MS/MS analysis with a stable‐isotope internal standard and colorimetric assays confirmed the elevated fumarate levels in Pck1 AKO adipose tissue (Figure 5g; Figure S5a). Moreover, exogenous monomethyl fumarate (MMF) supplementation exacerbated a senescence‐associated phenotype in 3T3‐L1 adipocytes, which was accompanied by a marked increase in the expression of p21 and p16 proteins (Figure 5h) and SASP factors (Figure S5h–n). These findings indicate that Pck1 deficiency accelerates aging‐associated metabolic reprogramming, with a pronounced impact on the TCA cycle in adipose tissue.

To explore whether metabolic flux alterations are associated with Pck1 deficiency, stable‐isotope tracing metabolomics technology was performed using [U‐^13^C] glutamine as a tracer (Figure 5i). We administered 2 mM [U‐^13^C]‐glutamine to the differentiated Pck1 KO and control adipocytes and measured the ^13^C‐labeled metabolites. The results showed that Pck1 deficiency did not affect glutamine uptake (Figure 5j). Despite it not altering the m + 2 isotopologues of α‐ketoglutarate (α‐KG), Pck1 deficiency resulted in noticeable increases in the m + 2 isotopologues of citrate, succinate, fumarate, malate, and oxaloacetate (OAA) (Figure 5k–p). Concurrently, the metabolism of [U‐^13^C]‐glutamine to glucose 6‐phosphate (G6P) m + 2 and fructose 1,6‐bisphosphate (F1,6‐BP) m + 2 was significantly decreased in Pck1‐deficient adipocytes (Figure 5q,r), indicating an impaired glyceroneogenic flux. Collectively, these findings indicate that Pck1 deficiency impedes the metabolic flux of TCA cycle intermediates toward glyceroneogenic precursors, leading to the accumulation of TCA cycle intermediates.

### 2.6 Deletion of Pck1 Disrupts Mitochondrial Homeostasis in Adipocytes

**FIGURE 6:**
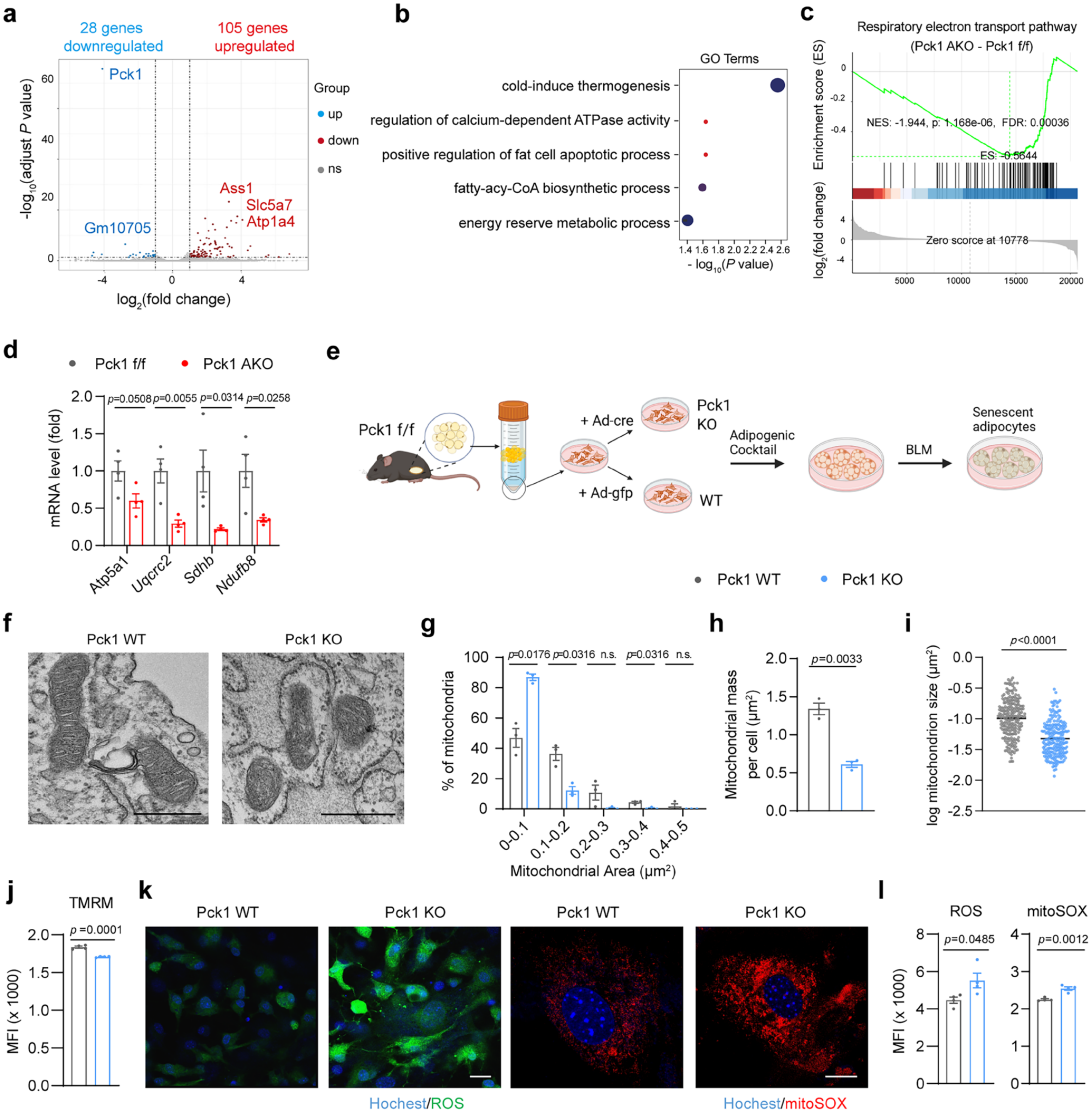
Pck1 deficiency leads to mitochondrial dysfunction in adipocytes. (a) Volcano plot representing differential genes in gWAT transcriptomes (Pck1 AKO versus Pck1 f/f) with |fold change| > 2, FDR < 0.05. (b) GO enrichment analysis to show mitochondrial‐related processes that were significantly enriched. (c) Gene Set Enrichment Analysis (GSEA) plot of enrichment in respiratory electron transport pathway (Reactome database, R‐HSA‐611105). (d) RT‐qPCR analysis of mitochondrial mRNA expression in mature adipocyte of 12‐month‐old Pck1 AKO mice and control mice. (e) Schematic diagram depicting the experimental procedure for the in vitro differentiated adipocyte model (Schematic created with BioRender.com. Agreement number: ZC28ATR6XD). (f) Transmission electron microscopy (TEM) images of mitochondria from Pck1 KO or WT adipocytes (scale bars, 500 nm). (g–i) Quantification analysis of mitochondrial size and mass from individual images. (j) Mitochondrial membrane potential was measured by TMRM staining using flow cytometry (*n* = 4). (k) Total reactive oxygen species (ROS) were measured with CM‐H2DCFDA and mitochondrial superoxide was measured with MitoSOX red, and cells were imaged using the Leica SP8 confocal microscope (scale bar, 20 μm). (l) Fluorescence intensity analysis of total ROS, and MitoSOX using flow cytometry with cells pre‐treated with BLM (*n* = 4). Data are mean ± SEM. Statistical analyses through the unpaired two‐tailed Student's *t*‐test (d–i).

Fumarate accumulation has been reported to be involved in mitochondrial damage in macrophages and renal cell carcinoma (Hooftman et al. 2023; Zecchini et al. 2023). Indeed, we observed that exogenous MMF supplementation in 3T3‐L1 adipocytes increased the mitochondrial reactive oxygen species (mtROS) levels and decreased mitochondrial membrane potential (Figure S5b). Based on this observation, we proposed that Pck1 deficiency caused mitochondrial dysfunction in adipocytes. To validate this hypothesis, we performed RNA‐seq analysis on Pck1‐deficient adipose tissue. Transcriptomic analysis identified 133 differentially expressed genes (DEGs), with 105 genes upregulated and 28 genes downregulated in gWAT of Pck1 AKO mice compared to Pck1 f/f mice (Figure 6a). Gene Ontology (GO) analysis of these 133 DEGs revealed significant enrichment in mitochondrial pathways, including cold‐induced thermogenesis and mitochondrial energy metabolism process (Figure 6b). Gene Set Enrichment Analysis (GSEA) further showed specific suppression of the respiratory electron transport pathway in gWAT of Pck1 AKO mice (Figure 6c). RT‐qPCR and WB analysis confirmed the reduced expression of mitochondrial proteins such as ATP synthase F1 subunit alpha 1 (Atp5a1), succinate dehydrogenase complex iron–sulfur subunit B (Sdhb), ubiquinol‐cytochrome C reductase core protein 2 (Uqcrc2), mitochondrially encoded cytochrome c oxidase I (Mtco1), NADH:ubiquinone oxidoreductase subunit B8 (Ndufb8) in 12‐month‐old PCK1 AKO mice gWATs (Figure 6d; Figure S5c).

To further investigate the effect of Pck1 deficiency on the mitochondrial dysfunction in adipocytes, we established a BLM‐induced senescent cell model using the differentiated adipocytes (Figure 6e). The cells were derived from SVFs of Pck1 f/f mice and then infected with adenovirus expressing Cre to induce Pck1 deficiency (Pck1 KO). Transmission electron microscopy (TEM) revealed a significant reduction in mitochondrial size and mass in senescent Pck1 KO adipocytes when compared to Pck1 f/f adipocytes infected with control adenovirus (Pck1 WT) (Figure 6f–i). In addition, Pck1 KO adipocytes showed altered mitochondrial cristae, which were characterized by abnormal shapes, a marked reduction in number, and disordered arrangement (Figure 6f). Flow cytometry revealed a reduced mitochondrial membrane potential in Pck1 KO adipocytes (Figure 6j), indicating mitochondrial depolarization. Moreover, Pck1 KO adipocytes displayed a substantial increase in reactive oxygen species (ROS) production in both cytoplasmic and mtROS levels (Figure 6k,l). These findings demonstrate that Pck1 deficiency disrupts mitochondrial energy metabolism homeostasis and induces excessive mitochondrial ROS production in adipocytes.

### 2.7 Pck1 Deletion in Adipocytes Promotes Cellular Senescence and Inflammatory Responses Through Fumarate‐Engaged cGAS/STING Signaling

**FIGURE 7:**
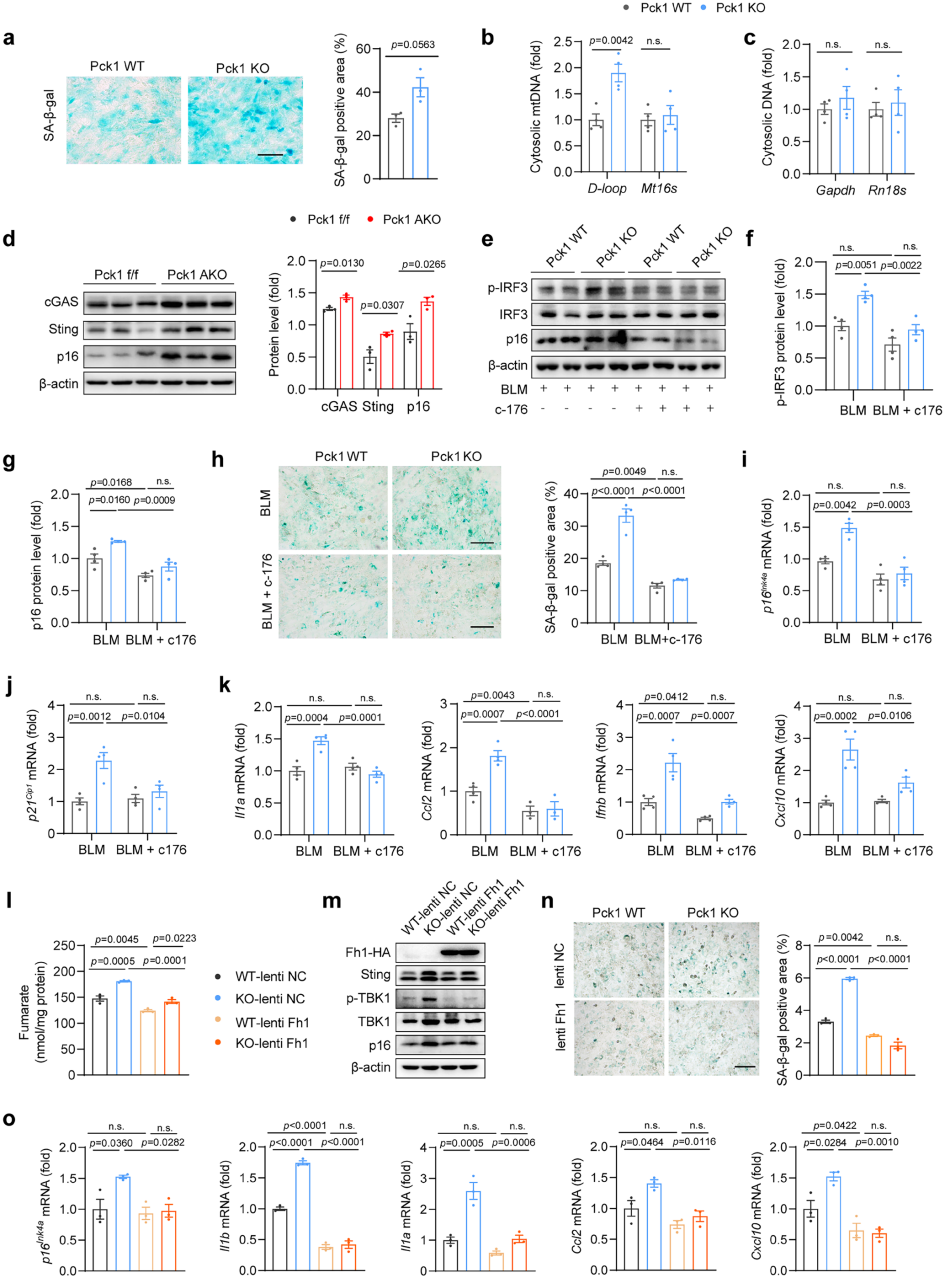
Pck1 deficiency promotes inflammaging via fumarate‐induced activation of mtDNA/cGAS/STING signaling. (a) Representative images of SA‐β‐gal staining with BLM‐treated Pck1 KO or WT adipocytes. Right, quantitative analysis of SA‐β‐gal positive area (*n* = 3). (b, c) Quantification of cytoplasmic DNA in BLM‐treated in vitro matured Pck1 WT and KO adipocytes by RT‐qPCR (*n* = 4). (d) Immunoblots of cGas, Sting, and p16 proteins in gWAT of middle age Pck1 AKO mice (*n* = 3). (e) Representative images of Western blot analysis in Pck1 WT or KO adipocytes after specific treatment. (f) Quantification of p‐IRF3 protein level related to total IRF3 (*n* = 4). (g) Quantification of p16 protein level (*n* = 4), β‐actin as loading control. (h) SA‐β‐gal staining of senescent WT or KO adipocytes treated with a Sting inhibitor c‐176 or vehicle‐treated control (scale bars, 200 μm). Quantification of SA‐β‐gal positive area (*n* = 4). (i‐k) RT‐qPCR analysis of *p16* ^*Ink4a*^, *p21* ^*Cip1*^, *Il1a*, *Ccl2*, *Ifnb*, and *Cxcl10* in SVF‐derived WT or KO adipocytes followed by c‐176 treatment. (l) Fumarate relative abundance in Pck1 WT or KO adipocytes after infection with lenti Fh1 or lenti NC (*n* = 3). (m) Protein levels of p16, Sting, and p‐TBK1 in Pck1 WT or KO adipocytes accompanied by the infection of lenti Fh1 or lenti NC. (n) Representative images and quantification of SA‐β‐gal staining with lenti Fh1 or lenti NC infected Pck1 KO or WT adipocytes (*n* = 4 in total) (scale bars, 200 μm). (o) RT‐qPCR analysis of the marker genes of aging and SASPs (*n* = 3). Data are mean ± SEM. Statistical analyses through the unpaired, two‐tailed Student's *t*‐test (a–d), or two‐way ANOVA with Tukey's HSD post hoc analysis.

Accumulation of ROS is known to cause damage to mtDNA and membranes (Richter 1992). Indeed, Pck1 KO adipocytes exhibited a significant increase in senescence‐associated markers (Figure 7a, Figure S4b–d) and enhanced mitochondrial DNA release (Figure 7b). Notably, the release of mitochondrial ribosomal DNA (mt16s) and genomic DNA remained unaltered, likely due to the heightened susceptibility of the D‐loop to ROS‐induced damage (Figure 7b,c). After confirming the purity of the cytosolic fraction by detecting marker proteins (Gapdh in supernatant; Tomm20 and Lamin A/C in pellet) (Figure S5d), we quantified mtDNA in the cytosol and confirmed its release from mitochondria. The elevated levels of cytoplasmic mtDNA and increased SASP factors in Pck1‐deficient cells prompted us to measure the downstream activation of the cGAS/STING signaling pathway. The results showed that a significant upregulation of both cGAS and STING protein levels was seen in Pck1 AKO mice, compared to Pck1 f/f mice (Figure 7d).

Given the established correlation between cGAS/STING signaling and cellular senescence (Guo et al. 2023), we sought to determine whether the activated cGAS/STING signaling mediates the exacerbating effect of Pck1 deficiency on adipocyte senescence. The results showed that treatment with the selective STING inhibitor c‐176 restored the levels of p‐IRF3, p‐STING, and p16 in Pck1 KO cells to those observed in Pck1 WT cells (Figure 7e–g; Figure S5e). In addition, c‐176 reduced SA‐β‐gal activity and normalized the mRNA expression levels of p16^Ink4a^ and p21^Cip1^ in Pck1 KO adipocytes to the WT controls (Figure 7h–j). Furthermore, pro‐inflammatory markers in Pck1‐deficient cells, including *Il1a*, *Ccl2*, *Ifnb*, and *Cxcl10*, were restored to baseline levels following c‐176 treatment (Figure 7k). These findings demonstrate that inhibition of STING‐dependent downstream signaling effectively reverses the senescence‐associated inflammatory phenotype induced by PCK1 deficiency in adipocytes, highlighting a critical role for cGAS/STING signaling in mediating the pro‐senescent effects of PCK1 loss.

To clarify whether fumarate accumulation mediates this inflammatory cascade in adipocytes, we investigated cGAS/STING signaling in MMF‐treated 3T3‐L1 adipocytes. Consistent with the observations in Pck1‐deficient cells, MMF treatment significantly increased cytoplasmic mtDNA abundance, accompanied by the upregulated expression of cGAS, STING, and SASP genes (Figure S5f–n). Pharmacologic inhibition of STING further confirmed a potential role of STING signaling in fumarate‐induced upregulation of inflammatory factors (Figure S5h–n). To determine whether fumarate mediates pro‐senescent effects of Pck1 loss on adipocytes, we employed lentiviral overexpression of fumarate hydratase (lenti Fh1) to reduce fumarate levels in Pck1 KO adipocytes (Figure 7l). The results showed that lenti Fh1 significantly attenuated the activation of the cGAS/STING pathway, along with the subsequent increase in SA‐β‐gal activity and SASP gene expression, caused by PCK1 knockout (Figure 7m–o). Collectively, these findings indicate that fumarate accumulation engages the mtDNA/cGAS/STING pathway and associated SASP induction, contributing to PCK1 deficiency‐induced adipocyte senescence and inflammation.

## 3 Discussion

**FIGURE 8:**
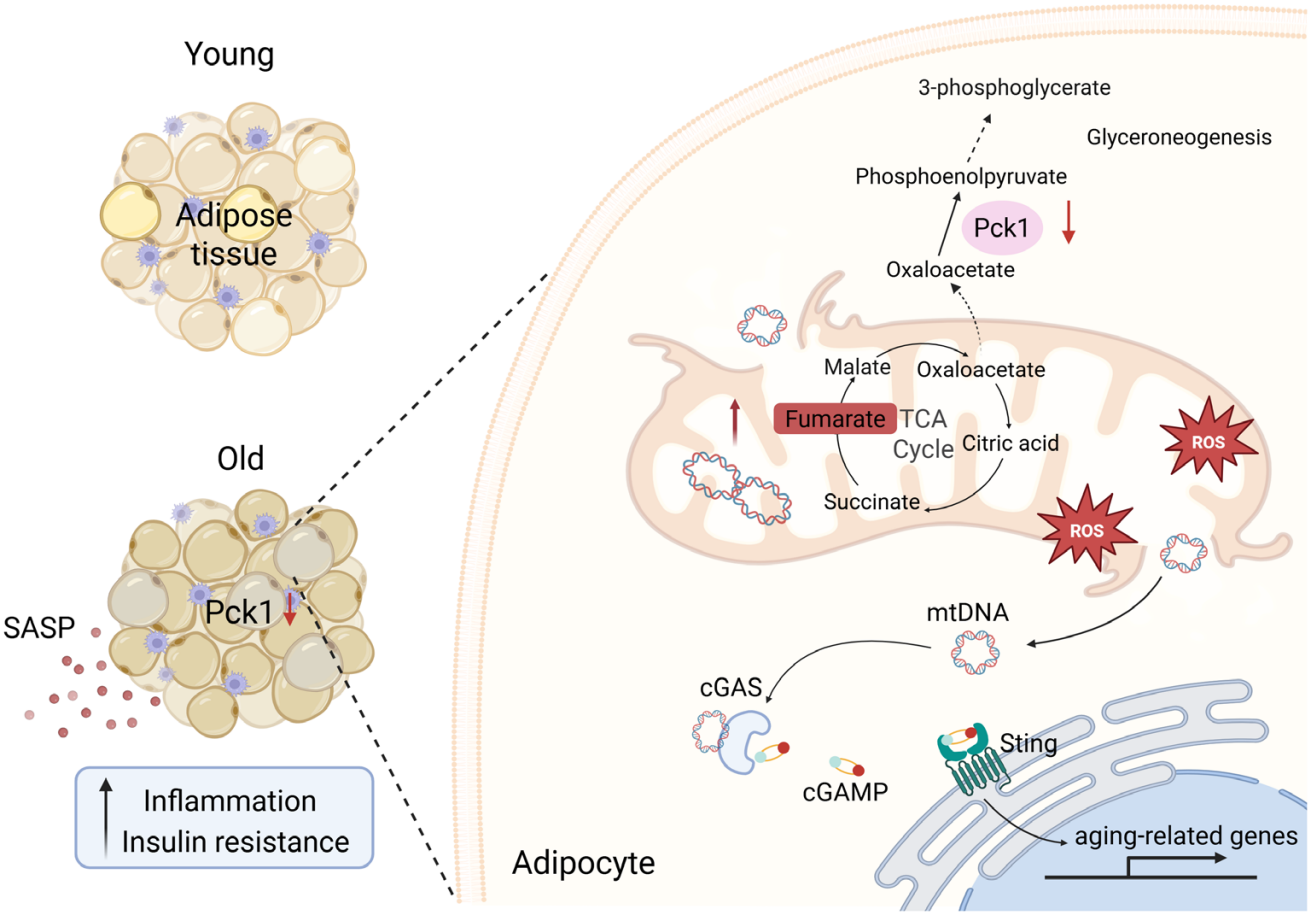
Graphic illustration to summarize insights into metabolic reprogramming in Pck1‐deficient adipocytes and the role of cGAS‐STING signaling in aging (Schematic created with BioRender.com. Agreement number: CQ28ATQQV2).

With global demographic shifts toward an aging population, extending health span and mitigating senescence‐related pathologies have emerged as fundamental research objectives (MacNee et al. 2014). Age‐related adipose tissue remodeling constitutes a significant contributor to systemic inflammaging, facilitating the pathogenesis of metabolic disorders in the elderly (Zamboni et al. 2021), but the mechanisms involved are still not well known. This study identifies a crucial role of Pck1 in controlling adipocyte senescence, adipose tissue inflammaging and associated systemic metabolic disorders. Pck1 deficiency impairs the cataplerotic flux, resulting in fumarate accumulation in adipocytes. This accumulation subsequently promotes the release of mtDNA and activation of cGAS/STING pathway‐induced immune responses, thereby contributing to inflammaging (Figure 8). Our study has uncovered a critical role of Pck1 in bridging metabolism, inflammation and aging in adipose tissues, and targeting Pck1 might be an effective strategy for the treatment of age‐related diseases.

The *Pck1* gene encodes the cytoplasmic isoenzyme of phosphoenolpyruvate carboxykinase (PEPCK‐C), which catalyzes the conversion of oxaloacetic acid (OAA) in the cytoplasm into phosphoenolpyruvate (PEP). Pck1 is critical for gluconeogenesis, glyceroneogenesis, and cataplerosis (Reshef et al. 1970; Reshef et al. 1969; Burgess et al. 2004). Pck1 deficiency in hepatocytes impairs TCA cycle intermediate efflux, leading to the excessive accumulation of metabolites, such as OAA and succinyl CoA (Xiang et al. 2021; Gou et al. 2023). These metabolites allosterically regulate enzymatic activity within the TCA cycle and induce metabolic shutdown (Beale et al. 2007; Owen et al. 2002). Pck1 ablation is also linked to mitochondrial oxidative stress and dysfunction, with altered mitoribosomal defects and GSH/GSSG ratios implicated (Verissimo et al. 2023; Hasegawa et al. 2023; Ma et al. 2018), though the precise mechanisms remain elusive. In this study, we used isotope tracer metabolomics analysis and found that Pck1 deficiency disrupted adipocyte cataplerosis, causing the accumulation of most intermediates in the TCA cycle, especially fumarate. Furthermore, we identified fumarate as a key mediator of Pck1 deficiency‐induced mitochondrial damage.

Published studies have demonstrated that polar metabolites in TCA cycle, such as fumarate, succinate, and α‐KG influence immune response through a “non‐metabolic” signal transmission (Pålsson‐McDermott and O'Neill 2025; Ryan et al. 2019). A growing body of evidence identifies the mtDNA/cGAS/STING signaling axis as a critical mechanism driving immune activation (Gulen et al. 2023; Chen and Xu 2023). Notably, fumarate accumulation has been shown to induce mtDNA release into the cytosol, triggering a type I interferon response via the cGAS/STING pathway in renal cell carcinoma (Zecchini et al. 2023). In the current study, we treated adipocytes with MMF or overexpressed fumarate‐metabolizing enzymes (Fh1 in rodents), confirming that the elevated fumarate levels led to mitochondrial damage and subsequent mtDNA release into the cytoplasm. Furthermore, treatment with STING inhibitors attenuated the effects of Pck1 deficiency or MMF incubation on the expression of SASPs and senescence‐related genes, indicating a critical role of the STING pathway in mediating the inflammatory and senescence‐associated responses induced by Pck1 deficiency or MMF treatment.

The accumulation of senescent cells is a well‐established hallmark of organ aging (van Deursen 2014), although their precise origin and consequence remain to be determined. Adipose tissue comprises of several cell populations including mature adipocytes, immune cells, progenitor cells, and endothelial cells that exhibit different responses to aging (Smith et al. 2021). Our research demonstrated that senescent cells of gWAT from Pck1 adipocyte‐specific knockout mice are primarily mature adipocytes rather than SVFs. Unlike the obesity‐ and hyperinsulinemia‐driven senescence model described by Li et al. (Li et al. 2021), our findings reveal a distinct age‐related increase in the transcripts for key senescence markers *p16* ^*Ink4a*^, *p21* ^*Cip1*^, and *p53* in isolated purified adipocytes. This finding aligns with prior studies showing that DNA damage repair‐induced senescence in mature adipocytes is critical for remodeling of adipose tissue and aging‐related metabolic complications (Lee et al. 2022; Liu et al. 2018). Notably, previous research in hepatic fibroblasts has linked the cGAS/STING/IRF3 signaling to the pRB/p16 axis, compelling evidence that cGAS/STING activation directly promotes cellular senescence (Wu et al. 2024). The age‐related decline in Pck1 expression in white adipose tissue triggers adipocyte senescence, which is also dependent on DNA damage‐induced cGAS/STING signaling.

Age‐related increases in VAT are associated with the infiltration of certain lymphocyte subsets, including macrophages, regulatory T cells, and B cells, contributing to inflammaging (Covarrubias et al. 2020; Bapat et al. 2015; Carter et al. 2018; Camell et al. 2019). We found that PCK1 deficiency enhanced macrophage infiltration in adipose tissue of both naturally aged and diet‐induced obese mice. Our findings revealed an elevated inflammatory secretory profile in Pck1‐deficient mature adipocytes, characterized by increased expression of pro‐inflammatory cytokines and chemokines, such as *Il6*, *Il1a*, *Ccl2*, *Ifnb*, and *Cxcl10*. Studies have shown that the activation of inflammatory signaling pathways, such as JAK/STAT, NF‐κB, and JNK, is critical for initiating the SASPs and establishing a pro‐inflammatory microenvironment in WAT (Zhang et al. 2023). Our results demonstrated that phosphorylation of IRF3 and TBK1, downstream of STING, serves as a key regulatory mechanism for SASP‐associated gene transcription. Pharmacological inhibition of STING significantly attenuated the mRNA expression of SASP factors and inflammatory mediators, which are known to promote macrophage migration.

With advancing age, VAT undergoes significant pathological changes, including hormone insensitivity, chronic inflammation, and cellular senescence, which collectively contribute to system metabolic disorders such as type 2 diabetes mellitus (Nguyen and Corvera 2024; Reyes‐Farias et al. 2021). Pck1 dysregulation in liver and adipose tissue has been implicated in the development of obesity and insulin resistance (Cadoudal et al. 2008; Rotondo et al. 2017; Yang et al. 2009). Our studies confirmed that Pck1 deficiency exacerbates insulin resistance in both naturally aging and diet‐induced obese mouse models. Additionally, Pck1‐deficient mice exhibited elevated serum lipid levels, further underscoring the metabolic impact of Pck1 on dysregulation.

Although this study elucidates the downstream effects of Pck1 downregulation in adipocyte senescence, the upstream mechanisms governing age‐related Pck1 expression decline remain unclear. Our preliminary analysis of single‐cell RNA‐seq data from adipocyte subset across age groups identified transcription factors ESRRG and ZFP354C as potential regulators, given their predicted binding to Pck1 promoter motifs and correlated expression patterns with Pck1 during aging. Determining whether Pck1 loss occurs via transcriptional, epigenetic, or post‐translational mechanisms will be a focus of our future research; identifying these upstream regulators is essential for understanding the metabolic aging network in adipose tissue. In addition, it remains unclear whether Pck1 deficiency elicits comparable synergistic effects across different adipose tissue depots and metabolic organs. To address this, future studies employing multi‐organ models are essential to elucidate the systemic consequences of adipose tissue‐specific Pck1 deficiency and its role in driving whole‐body inflammation and metabolic dysfunction.

In conclusion, our study demonstrates Pck1 deficiency in adipocytes as a pivotal factor in driving adipose tissue inflammaging. Our in vivo and in vitro findings show that fumarate‐linked mtDNA stress contributes to cGAS/STING activation and adipose inflammaging. These findings highlight novel therapeutic targets for mitigating adipose inflammation and age‐related metabolic diseases, offering significant potential to enhance metabolic health and longevity.

## 4 Methods

### 4.1 Mice

All mice were in a pure C57BL/6J background. Mice were housed at 22°C with 12 h of light–dark cycles and 50% humidity with free food (Chow diet, Jiangsu Xietong, 1010084) and water. The Adipoq‐cre mice were generously provided by Prof. Jinke Cheng (Hainan academy of medical sciences, Hainan medical university). We have authorized Cyagen (China) to employ CRISPR/Cas‐mediated genome engineering to generate the Pck1 floxed mice, whose exons 4–5 were selected as the conditional knockout region. Adipoq‐cre mice were crossed with Pck1 floxed mice to generate adipocyte‐specific Pck1 knockout (Pck1 AKO) mice. All mice were bred and maintained in specific pathogen‐free conditions. This study was approved by the Experimental Animal Center of Guangdong Medical University. We have complied with all relevant ethical regulations for animal use. For the HFD model, 10‐week‐old male Pck1 AKO or Pck1 f/f mice were randomly divided into two groups, subsequently fed a high‐fat diet (Research Diets, D12492) over a 16‐week period.

### 4.2 Glucose and Insulin Tolerance Tests

To assess glucose tolerance (GTT), mice were fasted overnight (17:30–9:30) before receiving d‐glucose (1.5 g/kg, Sigma, G8270) via intraperitoneal administration. For insulin sensitivity testing (ITT), mice were subjected to 4‐h fasting (10:00–14:00) followed by intraperitoneal insulin injection (0.8 U/kg, Yeasen 40112ES60). Blood glucose concentrations were monitored using an Ultra One glucometer at 15, 30, 60, 90, and 120 min intervals after treatment.

### 4.3 In Vivo Insulin Signaling

After overnight fasting, mice were injected intraperitoneally with insulin (0.8 U/kg, Yeasen 40112ES60) or matching saline control. Fifteen minutes later, mice were terminated and WAT tissues were immediately harvested and snap‐frozen in liquid nitrogen for subsequent analysis.

### 4.4 RNA Sequencing Analysis

RNA‐seq was performed by the Shanghai Applied Protein Technology Co. Ltd. (Shanghai, China). In brief, mRNA was concentrated using Oligo‐dT bead selection and fragmented. Library generation included first‐strand cDNA production from mRNA templates via random hexamer priming, then second‐strand cDNA isolation using AMPure XP bead purification. Subsequently, cDNA experienced end modification, 3′‐terminal adenine incorporation, and Illumina adapter ligation at the ends. RT‐qPCR enrichment was performed to obtain the final cDNA library. Library quality was assessed on an Agilent Bioanalyzer 4150 system, and sequenced on an Illumina Novaseq 6000/MGISEQ‐T7 instrument. Raw reads were processed in fastq format to remove low‐quality reads. In this step, Poly‐A tails and adaptor sequences, as well as low‐quality reads, were removed. After quality control, reads were mapped to the reference genome GRCm39 (GCA_000001635.9) using HISAT2 software. Gene counts and UMI counts were acquired by the feature Counts software. Based on gene length and read count, FPKM was estimated for each gene.

### 4.5 Senescence‐Associated β‐Galactosidase Staining

Detection of senescent cells was performed with the Senescence‐Associated β‐Galactosidase Staining Kit (Sigma, 94433). According to the manufacturer's protocol, WATs were first cut into pieces of equal size. Tissues or cultured cells were fixed in the fixation solution and incubated with a staining mixture solution at 37°C for 4 h to overnight.

### 4.6 WAT Histological Analysis

For WAT histology, formalin‐fixed paraffin‐embedded sections were stained with H&E and examined under a microscope. Both gWAT and iWAT slices were stained with Sirius Red to evaluate fibrosis. For immunofluorescence analysis, tissue sections underwent xylene deparaffinization and ethanol gradient rehydration to ddH_2_O. Antigen retrieval was performed by heating slides in 10 mM sodium citrate buffer for 15 min, followed by 30‐min blocking in PBS containing 5% BSA. Primary antibodies including F4/80 (1:400, Abcam ab6640) and Pck1 (1:400, Proteintech 66862‐1‐Ig) were applied overnight at 4°C as specified per experiment. Following triple PBS washing, secondary antibodies Alexa Fluor 488 Goat Anti‐Rat IgG (1:200, Abcam ab150165) and Alexa Fluor 488 Donkey anti‐Mouse IgG (1:200, Abcam ab150105) were incubated for 1 h at room temperature. After three PBS rinses, slides received DAPI counterstaining and were mounted with Anti‐Fade solution (Invitrogen, P36984). Imaging was conducted using a Leica SP8 confocal fluorescence microscope. A minimum of four cross‐sectional specimens per animal were analyzed and assessed. Quantification of positive regions was performed utilizing Image J software.

### 4.7 Transmission Electron Microscope

Adipocytes were grown on 6‐well plates and differentiated as described. Cells were digested with trypsin, then the pellet was collected and fixed in 1.5 mL centrifuge tubes with 2.5% glutaraldehyde solution at 4°C overnight. TEM was performed by the Sinoma Institute of Materials Research (Guang Zhou) Co. Ltd. The cells were postfixed in 1% osmium tetroxide for 2 h at room temperature. Subsequently, samples were rinsed in PBS and dehydrated using various concentrations of alcohol. Cells were excessively embedded in plastic and polymerized by heating at 70°C overnight. Ultra‐thin sections were generated by employing a Leica EM UC7 ultramicrotome, placed onto copper grids with lead citrate staining, and analyzed using an FEI Talos L120C field emission transmission electron microscope. Mitochondrial pictures of 15 cells were examined with ImageJ to quantify the area and distribution of all mitochondria.

### 4.8 Serum Lipid Measurements

Mice were fasted overnight and caged for 12 h in light–dark cycles with free water before sacrificed. The blood was collected and immediately centrifuged at 6000 rpm at 4°C for 15 min. TG, TC, and FFA levels were determined using commercial kits following the manufacturer's instructions (Nanjing Jiancheng Bioengineering Institute).

### 4.9 Isolation of Mouse Adipose SVFs

gWAT obtained from Pck1 f/f or Pck1 AKO mice was minced and digested in a 50 mL Falcon tube containing 5 mL digestion buffer [0.3% w/v collagenase II (Gibco, 17101015)], and incubated at 37°C for 30 min. After stopping the digestion by adding 15 mL 10% fetal bovine serum (FBS) (Gibco, 10378016) in DMEM (Hyclone, SH30243), the solution was filtered through a 70 μM cell strainer (Corning, 352350) and centrifuged at 400*g* for 10 min. Pelleted SVFs were collected for further experiments.

### 4.10 Adenovirus Production and Infection

Ad‐CRE or Ad‐GFP were generated following established protocols (Luo et al. 2007). Briefly, cDNA was cloned into pAdTrack‐CMV vectors, and viral construction utilized the pAd‐Easy system (Stratagene). Viral propagation occurred in 293A cells with subsequent purification via CsCl density gradient ultracentrifugation. Primary SVFs underwent adenoviral transduction at 100 pfu multiplicity of infection for 48 h before cell collection for subsequent analysis.

### 4.11 Adipocyte Differentiation

SVFs or 3T3‐L1 cells were cultured in complete medium (DMEM medium containing 2 mM L‐glutamine, 100 units/mL penicillin, 10 μg/mL streptomycin, and 10% FBS). Adipogenic differentiation was initiated using differentiation medium (complete medium containing 5 μg/mL insulin, 0.5 mM IBMX, 0.5 μM rosiglitazone, 2 μM dexamethasone, 125 μM indomethacin). Every 3 days, wash the cells and switch to a maintenance medium (complete medium containing 5 μg/mL insulin and 0.5 μM rosiglitazone). All chemicals for cell culture were obtained from Sigma‐Aldrich.

### 4.12 Senescence‐Inducing Agent Treatment to Cells

SVF‐derived adipocytes were treated with 50 μM Bleomycin (MCE, HY‐108345) in DMEM for 12 h, then cultured for the next 72 h with complete culture medium. For 3T3‐L1, MMF powder (MCE, HY‐W019696) was resuspended in DMSO at a concentration of 500 mM, and then added to the culture medium at the specified concentration. For prolonged treatments, the medium was refreshed every 48 h.

### 4.13 ROS and mitoSOX Detection

To detect cellular or mitochondrial ROS levels when alive, cells were seeded on a confocal dish and incubated with 10 μM DC‐FHDA (Biyotime, S0033S) or 1 μM MitoSOX (Thermo Fisher Scientific, M36009) and 10 ng/mL nuclear staining dye Hoechst‐33342 (Invitrogen, R37165) in a 37°C, 5% CO_2_ incubator for 20 min. Subsequently, rinse the cells with PBS and capture images using a confocal fluorescence microscope (Leica SP8).

### 4.14 Flow Cytometry of Adipocytes

Adipocytes were differentiated from SVFs in Pck1 AKO or Pck1 f/f mice for 6 days, and incubated with 10 μM CM‐H2DCFDA (Thermo Fisher Scientific, C6827) or 1 μM MitoSOX (Thermo Fisher Scientific, M36009) or TMRM mitochondrial membrane potential indicator staining (Thermo Fisher Scientific, I34361) and washed the cells with PBS before onboarding.

### 4.15 Western Blot Analysis

Mouse tissues and cells were homogenized and lysed in the RIPA Buffer (Beyotime Biotechnology, P0013B) supplemented with protease inhibitor cocktail (Beyotime Biotechnology, P1005). For adipose tissues, centrifuge at 12,000 rpm for 10 min at 4°C, and then aspirate the supernatant to remove lipids. The protein concentration was quantified by BCA Assay (Thermo Fisher, 23227). Primary antibodies used were Pck1 (1:1000, CST, 12940), p21 (1:500, Santa cruz, sc‐6246), p16 (1:500, Santa cruz, sc‐1661), p‐AKT (T308) (1:1000, CST, 2695), AKT (1:1000, CST, 4685), β‐Actin (1:2000, CST, 4970), cGAS (1:1000, CST, D1D3G), p‐STING (1:1000, CST, 72971), STING (1:1500, CST, 13647), p‐IRF3 (1:1000, CST, 29047), IRF3 (1:1000, CST, 4302), p‐TBK1 (1:1000, CST, 5483), TBK1 (1:1000, CST, 3504), Tomm20 (1:2000, CST, 42406), Lamin A/C (1:2000, CST, 2032), GAPDH (1:2000, CST, 97166), Total mitochondrial protein cocktail (1:1000, Abcam, ab110413), and incubated at 4°C overnight. The secondary antibodies used were incubated at room temperature for 1 h, blots were imaged by Amersham ImageQuant 800 Protein Blotting Imaging System. Gray values were measured using ImageJ software.

### 4.16 Quantification of mtDNA Release in Cytosolic Extracts

Quantification of mtDNA content was performed as previously described (Zhou et al. 2024). In brief, cells were collected and divided equally into two aliquots. One aliquot was resuspended in a digitonin buffer [150 mM NaCl, 50 mM HEPES pH 7.4, and 30 mg/mL digitonin (Selleck, E1321)]. The homogenized samples were placed on a rotator for 30 min at 4°C, then centrifuged at 16,000 g for 30 min. The supernatant (cytosolic DNA) was utilized for RT‐qPCR. The other aliquot was isolated in DNA extraction buffer (100 mM Tris–HCl, pH 8.5, 5 mM EDTA, pH 8.0, 0.2% SDS, 200 mM NaCl, 100 μg/mL proteinase K), and then incubated at 55°C for 4 h. Total DNA was extracted and amplified using primers specific for the mitochondrial genes (*D‐loop*, *Mt18s*) or nuclear genes (*Gapdh*, *Rn18s*), and normalized to the total DNA for each sample. The primer sequences can be found in Table S1.

### 4.17 RNA Preparation and RT‐qPCR

Total RNA was harvested using Trizol (Invitrogen, 15596026) according to the manufacturer's protocols. Then cDNA was generated with a PrimeScript RT reagent Kit (Takara, RR037). Quantitative PCR was performed using the SYBR Green Mix (Yeasen, 11202ES) in a QuantStudio 5 Real Time PCR System. The messenger RNA levels were normalized to 18S rRNA or β‐actin. The specific primer sequences were listed in Table S2.

### 4.18 Metabolite Labeling and Measurement by LC–MS/MS

After sacrifice, the adipose tissues were snap‐frozen in liquid nitrogen and stored at −80°C until analysis. The untargeted metabolomics was performed by Shanghai Applied Protein Technology Co. Ltd. (Shanghai, China). The samples were separated using a Vanquish LC ultra‐high performance liquid chromatography system and then analyzed by mass spectrometry using an Orbitrap Exploris 480 mass spectrometer (Thermo Fisher Scientific), with detection in electrospray ionization (ESI) positive and negative ion modes, respectively. The differentially expressed metabolites were filtered with a variable importance in projection value (VIP) > 1, and *p* value < 0.05 was considered to indicate statistically significant results. For fumarate detection, gWAT were obtained from 12‐month‐old mice. Metabolites were extracted from samples using acetonitrile: water (1:1) and derivatised with 3‐Nitrophenylhydrazine. Fumarate level was analyzed on a UPLC (Shimadzu 30 ad‐QTRAP 6500+), and d4‐fumaric acid (Cambridge Isotope Laboratories) was used as an internal standard for quantitation.

### 4.19 Determination of Cellular Fumarate

SVFs were first infected with Ad‐CRE or Ad‐GFP for 48 h, followed by induction of differentiation until Day 6. Then, lentivirus infection was conducted to overexpress Fh1 or GFP as control. Cells were washed three times with cold PBS, then the fumarate content of adipocytes was quantified using a Fumarate assay kit (Abcam, ab012516) following the manufacturer's instructions.

### 4.20 Lenti Virus Infection

Lentivirus overexpression mouse Fh1 and the control lentivirus overexpressing eGFP were all obtained from HanYi Biosciences Inc. (Guangzhou, China). Pck1 KO or WT adipocytes were infected by lentivirus with an MOI of 50, then the cells were cultured for 48 h before the next step.

### 4.21 Stable [U‐ ^13^C] Isotope Tracer Metabolomics

For isotopic labeling, cells were cultured in DMEM without glutamine (Gibco, 11054020) supplemented with 2 mM [U‐^13^C]‐glutamine (Cambridge Isotope Laboratories, CLM‐1822‐H), and 10% FBS for 24 h. To extract intracellular metabolites, cells were washed with cold PBS, then quenched with cold 80% methanol. The sample was centrifuged at 12,000 rpm and 4°C for 15 min. Transfer the clean supernatant to a new tube and evaporate it using a centrifugal concentrator. The dried extracts were reconstituted in a 5% acetonitrile aqueous solution and subsequently analyzed using LC–MS, and metabolite levels were normalized to the internal standard following established protocols.

### 4.22 Statistics Analysis

The data are presented as mean ± SEM, and statistical analysis was conducted using GraphPad Prism 8 Software. Data were analyzed using parametric tests as indicated: two‐tailed Student's *t*‐test for two‐group comparisons; one‐way ANOVA with Tukey's multiple comparison test for comparisons among three or more groups under a single independent variable; two‐way ANOVA with Tukey's HSD post hoc analysis for comparisons involving two independent variables. Where applicable, multiple *t*‐tests were corrected for false discovery rate (FDR) using the Benjamini‐Hochberg method. Sample sizes (*n* values) are provided in corresponding figure legends.

## Author Contributions

**Yiting Lei:** methodology, investigation, formal analysis, writing – original draft. **Meng Yang:** methodology, investigation, formal analysis, funding acquisition, writing – original draft. **Xiaoyun Jiang:** methodology, investigation. **Yujie Zhang:** methodology, investigation. **Yuzhi Chen:** methodology, investigation. **Weiheng Xie:** investigation. **Weihong Qin:** methodology, investigation. **Qihui Dai:** investigation. **Xiuqin Deng:** investigation. **Xiaojun Zhang:** investigation. **Zhongjun Zhou:** writing – revise. **Gonghua Huang:** funding acquisition, writing – revise, project conception, supervision. **Xinguang Liu:** funding acquisition, project administration, resources, supervision.

## Funding

This work was supported by the National Natural Science Foundation of China, 32341004, 81671399, 81971329, 82200821. Basic and Applied Basic Research Foundation of Guangdong Province, 2024A1515012922, 2021B1515130004, 2023A1515012838.

## Conflicts of Interest

The authors declare no conflicts of interest.

## Supporting information


**Table S1:** Primers sequence of mtDNA or genomic DNA used in this study.
**Table S2:** Primers sequence used in this study.

**Figure S1:** Pck1 deficiency also accelerates inflammaging in iWAT.
**Figure S2:** Normally aged mice displayed adipocyte senescence and disrupted lipid‐glucose homeostasis.
**Figure S3:** Pck1 AKO mice exhibited exacerbated insulin resistance.
**Figure S4:** No detectable off‐target effects observed for Adipoq‐cre.
**Figure S5:** MMF treatment induces mitochondrial dysfunction and inflammaging in adipocytes.

## Data Availability

The accession number for the non‐targeted metabolomics raw data reported in this paper is NGDC (https://ngdc.cncb.ac.cn): PRJCA055398, and the RNA‐seq raw data is NGDC: PRJCA055464.
