# Supplementary material for: Pck1 Deficiency Drives Mitochondrial Dysfunction and Cellular Senescence in Adipocytes
Source: Aging Cell. 2026 Mar 30;25(4):e70462. doi: 10.1111/acel.70462 (PMC13140901; doi:10.1111/acel.70462)
Supplement: Supplementary file 1 — Table S1: Primers sequence of mtDNA or genomic DNA used in this study. Table S2: Primers sequence used in this study. [file ACEL-25-e70462-s001.pdf]

Table S1. Primers sequence of mtDNA or genomic DNA used in this study

Name	Forward 5'-3'	Reverse 5'-3'
D-loop	AATCTACCATCCTCCGTGAAACC	TCAGTTTAGCTACCCCCAAGTTTAA
mt16s	CACTGCCTGCCCAGTGA	ATACCGCGGCCGTTAAA
Gapdh	ACCTGACTTTTAAGAGCAACTGGG	GGTGGTCCAGGGTTTCTTACTC
Rn18s	GTAACCCGTTGAACCCCATT	CCATCCAATCGGTAGTAGCG

Table S2. Primers sequence used in this study

Name	Forward 5'-3'	Reverse 5'-3'
p16Ink4a	CGCAGGTTCTTGGTCACTGT	TGTTCACGAAAGCCAGAGCG
p21Cip1	CCTGGTGATGTCCGACCTG	CCATGAGCGCATCGCAATC
Pck1	CTGCATAACGGTCTGGACTTC	CAGCAACTGCCCGTACTCC
p53	CCGACCTATCCTTACCATCATC	AGGCACAAACACGAACCTCAA
β-actin	ATGCTCCCCGGGCTGTAT	CATAGGAGTCCTTCTGACCCAT
Il6	CTTCCATCCAGTTGCCTTCT	CTCCGACTTGTGAAGTGGTATA
Il1a	CCTTACACCTACCAGAGTGATT	AACCAAGTGGTGCTGAGATAG
Il1b	CAACCAACAAGTGATATTCTCCATG	GATCCACACTCTCCAGCTGCA
Ccl2	CCCACTCACCTGCTGCTAC	TTCTTGGGGTCAGCACAGA
Tnfa	CAGGCGGTGCCTATGTCTC	CGATCACCCCGAAGTTCAGTA
Cxcl10	TTTCTGCCTCATCCTGCTG	TCCCTATGGCCCTCATTCT
Cxcl12	CTCTGCATCAGTGACGGTAAA	CACAGTTTGGAGTGTTGAGGA
Ifnb	ATGGTGGTCCGAGCAGAGAT	CCACCACTCATTCTGAGGCA
Atp5a1	TTTGCCCAGTTTGGTTCTGAT	CCCGTACACCCGCATAGATAA
Uqcrc2	TGGCTCTGGTTGGACTTGGT	TTTCACCTCCACGGTATTTGG
Sdhb	CTGTCGAGGGGCACAGAC	CAACACCATAGGTCCGCACT
Ndufb8	GGCACGTGTTCCCTTCCTAC	CCGCTCCAGGTACAGATTATTGT
