# Supplementary material for: Pck1 Deficiency Drives Mitochondrial Dysfunction and Cellular Senescence in Adipocytes
Source: Aging Cell. 2026 Mar 30;25(4):e70462. doi: 10.1111/acel.70462 (PMC13140901; doi:10.1111/acel.70462)
Supplement: Supplementary file 2 — Figure S1: Pck1 deficiency also accelerates inflammaging in iWAT. Figure S2: Normally aged mice displayed adipocyte senescence and disrupted lipid‐glucose homeostasis. Figure S3: Pck1 AKO mice exhibited exacerbated insulin resistance. Figure S4: No detectable off‐target effects observed for Adipoq‐cre. Figure S5: MMF treatment induces mitochondrial dysfunction and inflammaging in adipocytes. [file ACEL-25-e70462-s002.doc]

**
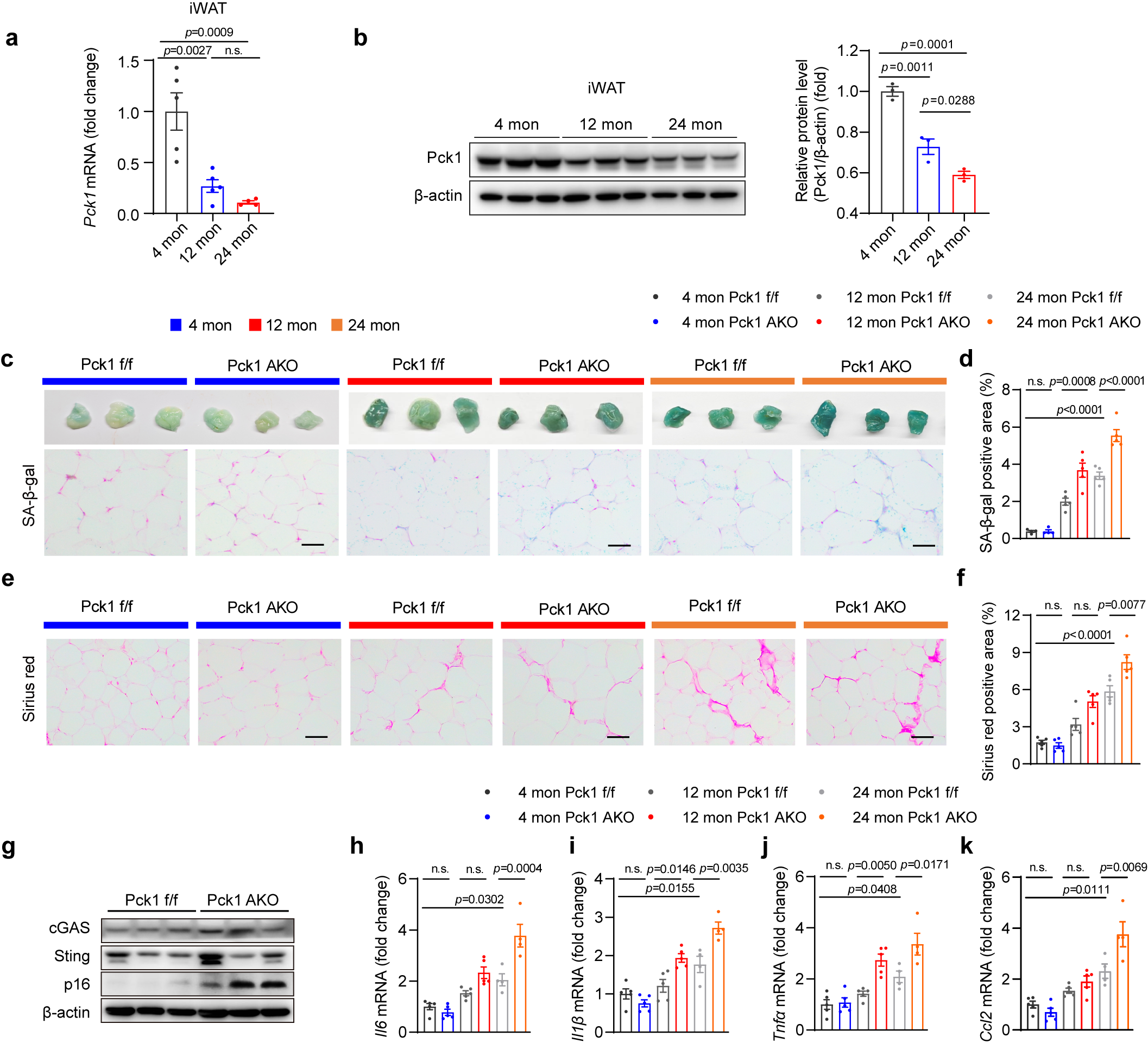
**

**Figure S1. Pck1 deficiency also accelerates inflammaging in iWAT.**

**a**, Detection of Pck1 mRNA level during the process of aging in iWAT (*n* = 5). **b**, Western blotting analysis of Pck1 protein levels of iWATs. β-actin as loading control (*n* = 3). **c**, **d**, SA-β-gal staining and quantitative analysis of iWAT in 4-month-old, 12-month-old, and 24-month-old Pck1 AKO and control mice (*n* = 5) (Scale bars, 50 µm). **e**, **f**, Representative images of adipose tissues with collagen staining (Sirius Red) (Scale bars, 50 µm). **g**, Western blotting of 12-month-old Pck1 AKO and control mice. **h-k**, RT-qPCR analysis of SASPs mRNA expression in iWAT from 12-mon and 24-mon old Pck1 f/f or Pck1 AKO mice. Relative to the young group. Data were analyzed statistically via one-way ANOVA or two-way ANOVA with Tukey’s HSD for multiple comparisons.

**
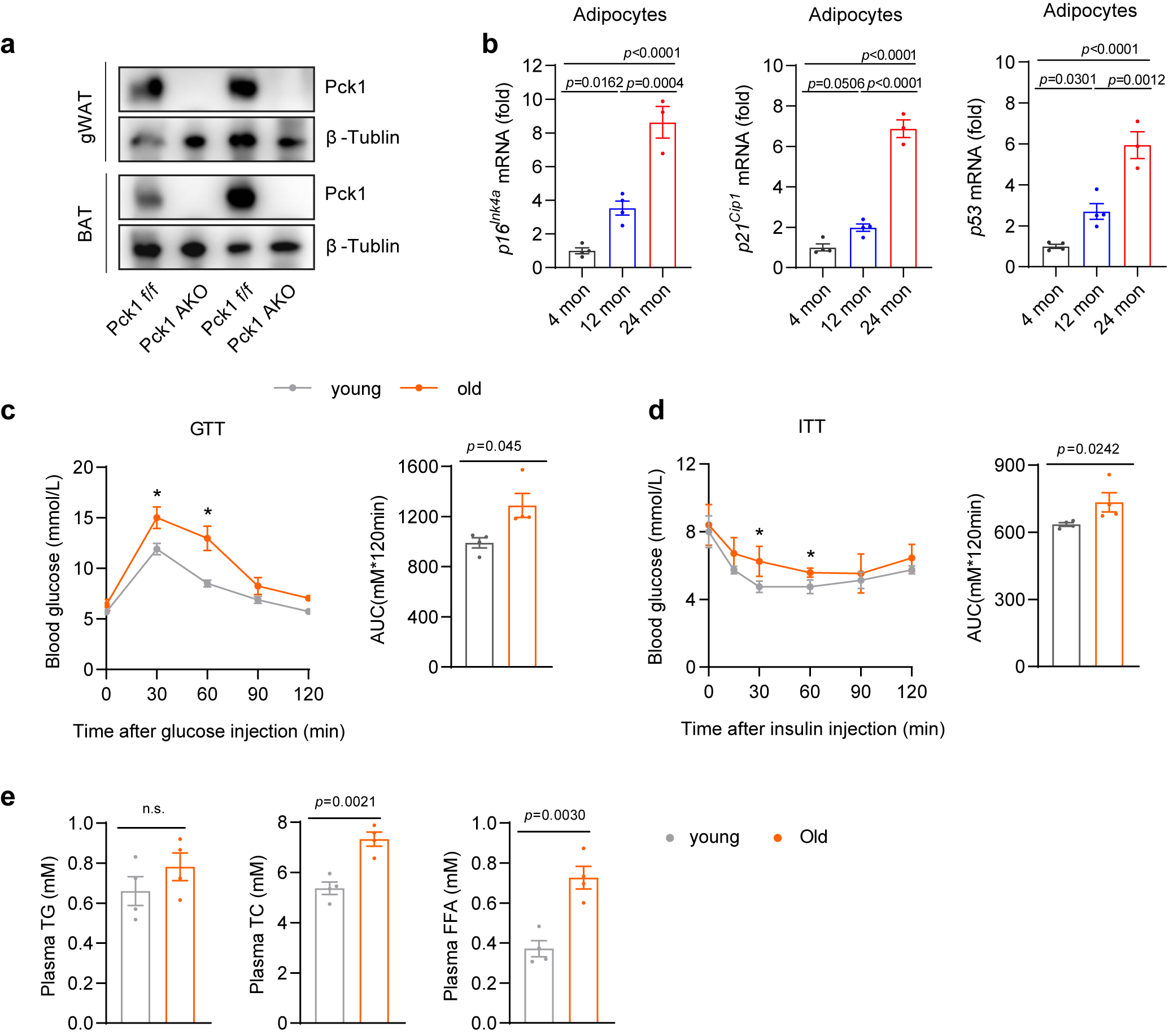
Figure S2. Normally aged mice displayed adipocyte senescence and disrupted lipid-glucose homeostasis.**

**a**, Western blot exhibits Pck1 level in adipose tissue of Pck1 AKO or Pck1 f/f mice. **b**, RT-qPCR analysis of senescence markers in SVFs (*n* = 4). **c**, **d**, GTT and ITT were performed on 4-month-old or 24-month-old wild-type mice (*n* = 4). **e**, Plasma TG, TC, and FFA levels of 4-month-old or 24-month-old wild-type mice (*n* = 4). Data were analyzed statistically via one-way ANOVA (**b**) or Student’s *t* test (**c-e**). * FDR< 0.05 and ** FDR < 0.01 by Student’s *t* test with Benjamini-Hochberg method for multiple comparisons.


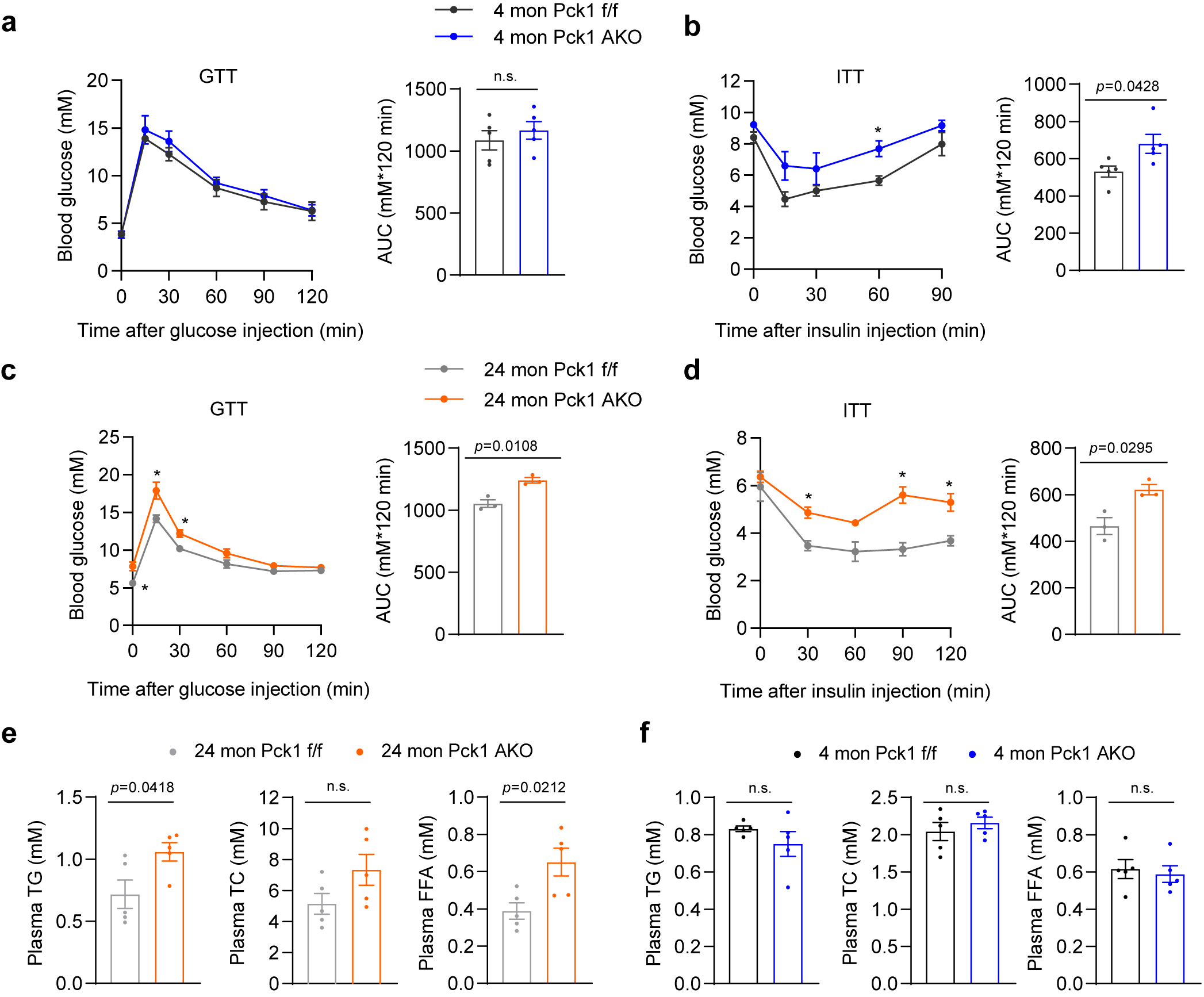


**Figure S3. Pck1 AKO mice exhibited exacerbated insulin resistance.**

**a**-**d**, GTT and ITT were performed on 4-month-old mice (**a**, **b**) or 24-month-old mice (**c**, **d**). **e**, **f**, Plasma TG, TC, and FFA levels of 24-month-old Pck1 AKO and f/f mice (**e**) or 4-month-old Pck1 AKO and f/f mice (**f**). Data were analyzed statistically via unpaired two-tailed Student’s *t* test with Benjamini-Hochberg method for multiple comparisons. * FDR< 0.05 and ** FDR < 0.01.


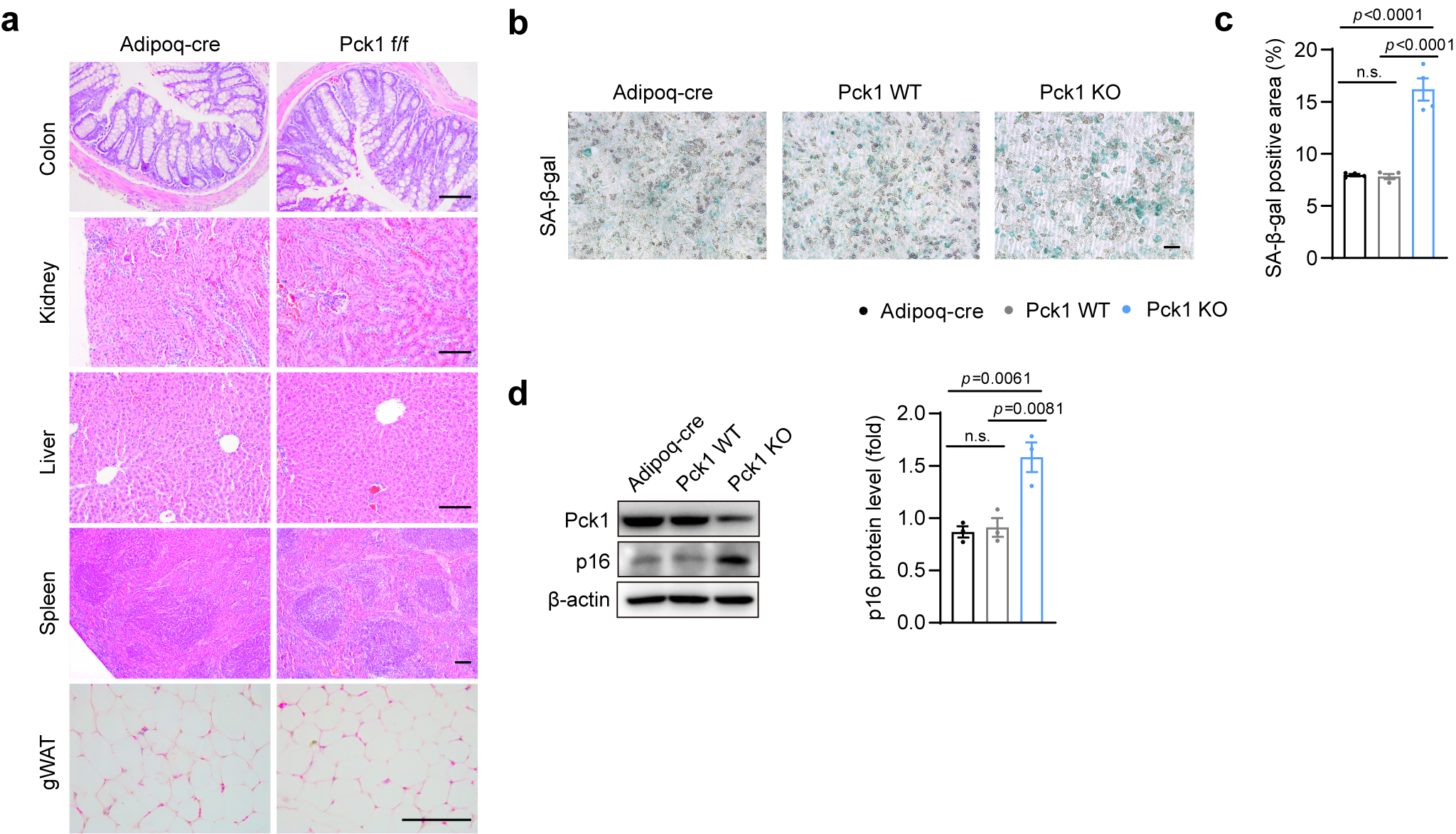


**Figure S4. No detectable off-target effects observed for Adipoq-cre.**

**a**, H&E staining of colon, kidney, liver, spleen, gWAT of 6-week-old Adipoq-cre and Pck1 f/f mice. **b**, SA-β-gal staining of senescent adipoq-cre, Pck1 WT, and KO adipocytes. **c**, Quantification of SA-β-gal positive area (*n* = 4). **d**, Representative images and quantification of Western blot analysis in Adipoq-cre, Pck1 WT, and Pck1 KO adipocytes (*n* = 4). Scale bar, 100 μm. Data are mean ± SEM. Statistical analyses through the one-way ANOVA with Tukey’s HSD post hoc analysis.

**
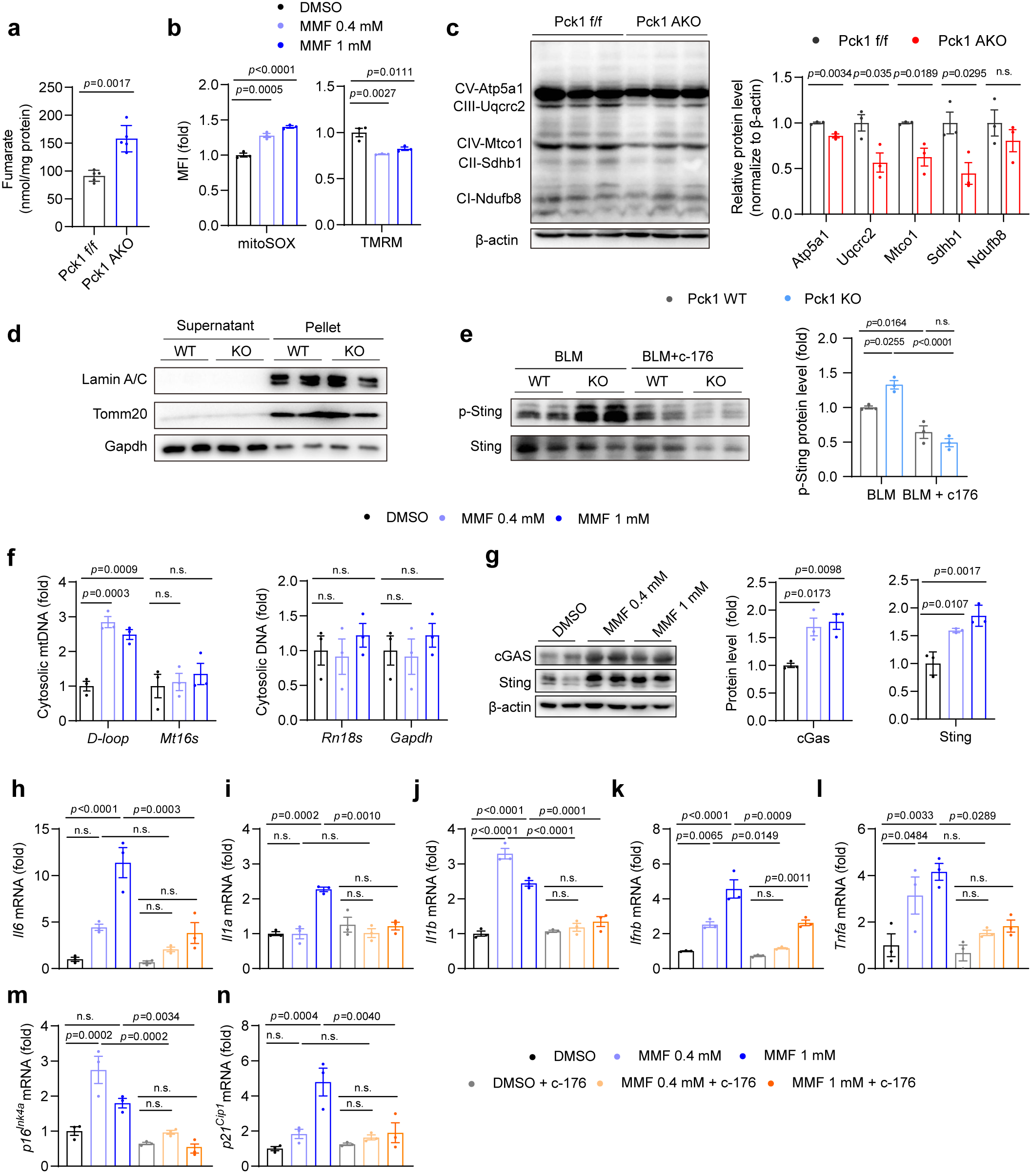
Figure S5. MMF treatment induces mitochondrial dysfunction and inflammaging in adipocytes.**

**a,** Detection of fumarate level in gWAT of 12-month-old Pck1 AKO or control mice, relative to total protein using a fumarate assay kit. **b**, Fluorescence intensity analysis of MitoSOX and TMRM using flow cytometry (*n* = 3). **c**, The protein level of mitochondrial proteins in gWATs of 12-month-old mice. **d**, Detection of cytosolic marker and mitochondrial markers as measurement controls. **e**, Western blot analysis of p-Sting level in Pck1 KO and WT adipocytes with BLM or c-176 treatment. **f**, Quantification of cytoplasmic mtDNA in MMF-treated 3T3-L1 adipocytes by RT-qPCR (*n* = 3). **g**, Western blot and quantitative analysis of the levels of cGAS and Sting induced by MMF (*n* = 3). **h-n**, RT-qPCR analysis of *Il6*, *Il1a*, *Il1b, Ifnb*, *Tnfa, p16^Ink4a^* and *p21^Cip1^* in differentiated 3T3-L1 adipocytes treated with MMF followed by c-176 treatment. DMSO treatment as controls. Data are mean ± SEM. Statistical analyses were conducted through the one-way ANOVA or two-way ANOVA with Tukey’s HSD for multiple comparisons.
